# Live cell tagging tracking and isolation for spatial transcriptomics using photoactivatable cell dyes

**DOI:** 10.1038/s41467-021-25279-y

**Published:** 2021-08-17

**Authors:** Alex S Genshaft, Carly G. K. Ziegler, Constantine N. Tzouanas, Benjamin E. Mead, Alex M. Jaeger, Andrew W. Navia, Ryan P. King, Miyeko D. Mana, Siyi Huang, Vanessa Mitsialis, Scott B. Snapper, Ömer H. Yilmaz, Tyler Jacks, Jeffrey F. Van Humbeck, Alex K. Shalek

**Affiliations:** 1 Institute for Medical Engineering & Science, MIT, Cambridge, MA USA; 2 Department of Chemistry, MIT, Cambridge, MA USA; 3 Koch Institute for Integrative Cancer Research, MIT, Cambridge, MA USA; 4 The Ragon Institute of MGH, MIT and Harvard, Cambridge, MA USA; 5 Broad Institute of MIT and Harvard, Cambridge, MA USA; 6 Harvard-MIT Program in Health Sciences and Technology, Harvard Medical School, Cambridge, MA USA; 7 Department of Immunology & HMS Center for Immune Imaging, Harvard Medical School, Boston, MA USA; 8 Division of Gastroenterology, Hepatology, and Nutrition, Boston Children’s Hospital, Boston, MA USA; 9 Division of Gastroenterology, Brigham and Women’s Hospital, Boston, MA USA; 10 Department of Biology, Massachusetts Institute of Technology, Cambridge, MA USA; 11 Department of Pathology, Massachusetts General Hospital and Harvard Medical School, Boston, MA USA; 12 Howard Hughes Medical Institute, Chevy Chase, MD USA; 13 Department of Chemistry, University of Calgary, Calgary, AB Canada

**Keywords:** RNA sequencing, Assay systems, Transcriptomics, Multicellular systems

## Abstract

A cell’s phenotype and function are influenced by dynamic interactions with its microenvironment. To examine cellular spatiotemporal activity, we developed SPACECAT—Spatially PhotoActivatable Color Encoded Cell Address Tags—to annotate, track, and isolate cells while preserving viability. In SPACECAT, samples are stained with photocaged fluorescent molecules, and cells are labeled by uncaging those molecules with user-patterned near-UV light. SPACECAT offers single-cell precision and temporal stability across diverse cell and tissue types. Illustratively, we target crypt-like regions in patient-derived intestinal organoids to enrich for stem-like and actively mitotic cells, matching literature expectations. Moreover, we apply SPACECAT to ex vivo tissue sections from four healthy organs and an autochthonous lung tumor model. Lastly, we provide a computational framework to identify spatially-biased transcriptome patterns and enriched phenotypes. This minimally perturbative and broadly applicable method links cellular spatiotemporal and/or behavioral phenotypes with diverse downstream assays, enabling insights into the connections between tissue microenvironments and (dys)function.

## Abstract (web summary)

Spatial transcriptomics aims to pair omic data with tissue structure. Here the authors report Spatially PhotoActivatable Colour Encoded Cell Address Tags (SPACECAT) to track and isolate live cells by location; this enables spatially informed downstream assays like scRNA-seq and flow cytometry.

## Introduction

From descriptions of gross anatomical features to characterization of subcellular organelles, modern in situ profiling methods have enabled insights into the organizational principles of biological structures. Next-generation single-cell genomic technologies hold great promise for discoveries in health and disease; however, they are typically performed on dissociated cells and thus lack information regarding cellular location, morphology, microanatomy, and physiological context.

Current strategies for matching -omic measurements with spatial resolution rely on genetic models^1–5^, fixed or frozen samples^6–8^, or computational inference^9,10^. Approaches that depend on genetic modifications facilitate dynamic characterizations in model systems but are not applicable to human clinical samples and can be labor intensive to create and maintain. Methods reliant on fixed or frozen samples, meanwhile, offer static snapshots of complex systems and can be applied to nearly any tissue type, yet they are not compatible with all downstream measurements (e.g., biophysical assays) and offer limitations in some high-throughput genomic assays (e.g., due to RNA degradation or input cell number). Similarly, computational methods rely on a priori knowledge of sample structure, which is possible in many healthy tissues but cannot always be assumed in biological systems or heterogeneous clinical samples.

To address these shortcomings, we introduce SPACECAT (Spatially PhotoActivatable Color Encoded Cellular Address Tags), a method for tagging and tracking cells from user-defined regions, enriching for observable features, and isolating cells for arbitrary downstream processing. We designed SPACECAT to be minimally invasive and compatible with a diverse set of input samples and to yield live cells that have been tagged in a user-designated fashion encoding prior tissue location, morphology, and/or other observable behaviors. In SPACECAT, a biological sample is iteratively immersed in media containing photocaged dyes, and specific areas of interest are illuminated with a spatially controlled near-ultraviolet (near-UV) light source. The near-UV light uncages the dye(s), generating fluorescent molecules in situ within cells in the photoactivated regions. These fluorescent cells can then be tracked over time and isolated via flow sorting, enabling arbitrary combination of imaging modalities with biophysical, functional, or genomic assays, all while retaining registry of user-tagged cells.

Here we apply SPACECAT to two-dimensional (2D) in vitro cultures, three-dimensional (3D) human intestinal organoids, and mouse tissue sections across health and disease. Through these example applications, we validate its spatial selectivity and precision via comparisons with known structural phenotypes and apply it to a lung tumor mouse model to further support hypotheses of spatial heterogeneity in the tumor-immune microenvironment. Crucially, SPACECAT can be incorporated into pre-existing sample processing pipelines, does not require complex chemistry to be performed upon samples, and can be applied to samples regardless of donor, species, or tissue of origin. Thus, SPACECAT holds promise to link microanatomical spatial knowledge and dynamic cellular phenotypes with the unbiased molecular resolution of highly parallel single-cell assays.

## Results

### Nitroveratryloxycarbonyl (NVOC) caging of calcein carboxylate groups enables rapid, stable, spatially selective generation of fluorescent calcein within live cells

**Fig. 1:**
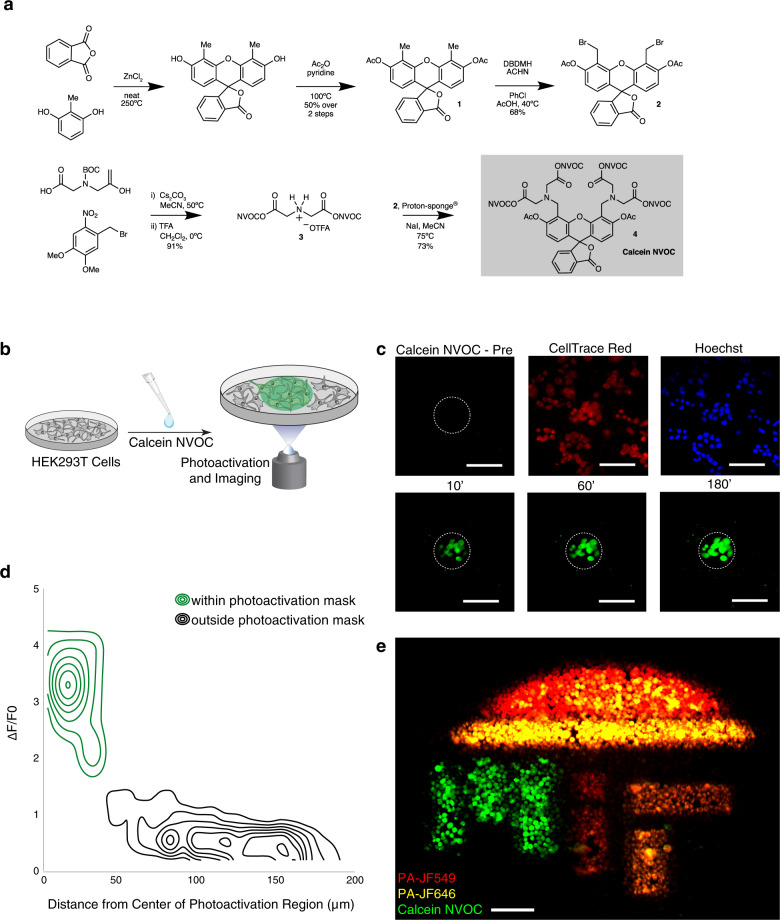
The SPACECAT protocol enables spatially precise, temporally stable fluorescence signals in arbitrary regions of interest. **a** Synthetic schema for calcein NVOC. **b** HEK293T cells were stained with calcein NVOC and imaged before photoactivation and afterwards for 3 h. Cells in schematic adapted with permission from ref. ^66^. **c** Representative time course images from a single field of view. Presented images of calcein NVOC were taken before photoactivation (calcein NVOC Pre), 10 min after (10’), 60 min after (60’), and 180 min (180’) after photoactivation. Images of CellTrace Red and Hoechst stains were taken 180 min after photoactivation. Dashed line: photoactivated region boundary; scale bar = 100 μm. See Supplementary Fig. 6a for quantitative time courses across *n* = 10 fields of view. **d** Contour plots representing the spatial distribution of fluorescence changes in photoactivated regions (green lines, *n* = 68 cells across 8 fields of view) and regions outside of photoactivation mask (black lines, *n* = 1347 cells across 8 fields of view). Δ*F*/*F*_0_ is calculated as a cell’s change in mean fluorescence 180 min after photoactivation, divided by the cell’s pre-photoactivation mean fluorescence. **e** Composite image of HEK293T cells stained and photo-uncaged within the “MIT” logo region. By sequential addition of 3 photoactivatable probes (calcein NVOC, PA-JF549, PA-JF646) and leveraging different photoactivation thresholds (10 s for calcein NVOC, 0.5 s for PA-JF549 and PA-JF646), 5-color encoding is achieved. Scale bar = 100 μm. Green: uncaged calcein NVOC; yellow: uncaged PA-JF646, red: uncaged PA-JF549. Multiplexed encoding scheme repeated a total of *n* = 2 times.

To arbitrarily tag live cells for tracking and isolation, we chose calcein as a scaffold to modify with NVOC photolyzable protecting groups (Fig. 1a, b and Supplementary Fig. 1a, b). As a commonly used viability dye, calcein enables determination of cell health as part of our cell tagging scheme without occupying an additional fluorescence channel^11–14^. Commercially available calcein acetoxymethyl (AM) has seven protected carboxylic acids, all of which must be deprotected for maximal fluorescence^15^. Six of these acids are protected as an AM esters, and the last is protected as a lactone. Upon entering the cell, the acids are deprotected and the molecule becomes fluorescent; the net negative charge that amasses during deprotection is thought to retain the fluorescent molecule in the cell (Supplementary Fig. 1b).

Here we photocaged calcein’s EDTA-like moieties by initially functionalizing the xanthene scaffold specifically at the 4’ and 5’ positions (Fig. 1a and Supplementary Fig. 1b)^16^. First, the xanthene scaffold (**1**, Fig. 1a and Supplementary Fig. 2) was synthesized with methyl groups in the 4’ and 5’ positions and acetyl-protected phenols with a 50% yield over two steps. This molecule was then brominated with 1,3-dibromo-5,5-dimethylhydantoin using 1,1’-azobis(cyclohexanecarbonitrile) as an initiator to form **2** with a 68% yield (Fig. 1a and Supplementary Fig. 3). In parallel, the NVOC-protected EDTA-like moiety (**3**, Fig. 1a and Supplementary Fig. 4) was synthesized by combining 2-nitro-4,5-dimethoxybenzyl bromide and *N*-Boc-iminodiacetic acid under basic conditions, removing excess alkylating agent with a silica plug, and finally removing the Boc protecting group in trifluoroacetic acid to deliver the trifluoroacetate salt of the desired amine in 91% yield. Immediately prior to reaction, the free amine of salt **3** was liberated and was then combined with **2** in the presence of sodium iodide and 1,8 bis(dimethylamino)naphthalene (i.e., Proton-sponge®) in dry acetonitrile until the reaction went to completion. After the removal of multiple impurities by four sequential precipitation/filtration steps (from MeCN; from CH_2_Cl_2_; from CH_2_Cl_2_/Et_2_O; from tetrahydrofuran (THF)/Et_2_O), the desired **4**, which we term calcein NVOC, was isolated by column chromatography in 73% yield (Fig. 1a and Supplementary Fig. 5).

As an initial test of calcein NVOC, we applied it to HEK293T cells in complete media and photoactivated user-defined regions of interest (ROIs) using a digital micromirror device (Fig. 1b). We evaluated different photoactivation protocols to balance strength of photoactivated fluorescence signal and phototoxicity (Supplementary Fig. 6a). Images taken before and after photoactivation revealed significant fluorescence increases, measurable immediately after active illumination and stable throughout a 3 h time course experiment, indicating both persistent cell tagging and preserved cell viability (Fig. 1c and Supplementary Fig. 6b, c). Furthermore, calcein NVOC’s fluorescence signals could distinguish photoactivated from non-photoactivated cells for at least 16 h after photoactivation, and there is literature support for calcein cell labeling for hours to days^17,18^. This temporal stability is critical for downstream experimental flexibility, enabling sample dissociation protocols, flow cytometry, and even subsequent functional or biophysical measurements following cell tagging. Alternately, cells can be tracked in situ, enabling experiments that combine behavioral readouts with spatial location and downstream measurements.

Moreover, fluorescence increases were specific to photoactivated ROIs as quantified by comparing the change in fluorescence inside vs. outside the photoactivated region (Fig. 1c, d and Supplementary Fig. 6b, c; *p* value < 0.0001, Student’s *T* test of 68 cells within vs. 1347 outside the photoactivated region, 8 fields of view; Supplementary Fig. 6d; *p* < 0.0001, Student’s *T* test of 1066 photoactivated cells across 11 fields of view vs. 2427 non-photoactivated cells across 5 fields of view). With calcein NVOC serving as a dual readout of cellular viability and spatial location, we sought to increase the multiplexing capability of our approach through two commercially available amine-reactive photoactivatable dyes^19^ with distinct fluorescence spectra (PA-JF549, PA-JF646; Supplementary Fig. 6e). Note that individual cells represent a lower bound on region size for an intracellular dye that does not target a specific protein, nucleic acid, or subcellular structure.

Finally, to demonstrate the spatial control and multiplexing capability of SPACECAT’s tagging procedure, we photoactivated HEK293T cells within a mask of MIT’s logo following sequential treatment with calcein NVOC, PA-JF549, and PA-JF646 (Fig. 1e, Supplementary Fig. 6f). Taking advantage of the different thresholds for photoactivation between distinct dyes, we uniquely tagged cells with five distinct color combinations and confirmed fluorescence was maintained through dissociation and flow cytometry (Supplementary Fig. 6f–n). This demonstrates the multiplexing capacity of calcein NVOC and an approach that enables simultaneous encoding of distinct microanatomical neighborhoods.

### Flow cytometry distinguishes calcein NVOC-tagged cells with high sensitivity

To demonstrate the specificity of both tagging and isolation of cells from desired photoactivated regions, we performed a species-mixing experiment where HEK293T (human) and NIH/3T3 (mouse) cells were co-cultured in a spatially structured manner (Supplementary Fig. 7a). After incubation with calcein NVOC, one of the two zones of cells (i.e., either mouse or human) was photoactivated, creating three conditions: (1) co-cultured NIH/3T3 and HEK293T, with photoactivated NIH/3T3, (2) co-cultured NIH/3T3 and HEK293T, with photoactivated HEK293T, and (3) co-cultured NIH/3T3 and HEK293T, both non-photoactivated. Cells were isolated by fluorescence-activated cell sorting (FACS), and calcein NVOC+ fluorescent cells from each experimental condition were individually sorted for single-cell RNA sequencing (scRNA-seq; Supplementary Fig. 7b)^20,21^. Single-cell libraries were aligned to a joint human and mouse reference genome, which identified each cell’s species of origin (Supplementary Fig. 7c). Of the 48 cells passing quality thresholds from the HEK293T-tagged condition, 45 aligned to the human transcriptome; from the NIH/3T3-tagged condition, 61 cells passed quality thresholds, 60 of which aligned to the mouse transcriptome. When compared with the non-photoactivated control, these distributions were significantly enriched for the desired species (*p* values = 0.026 and 5.5e−19 for human and mouse, respectively, Fisher’s exact test).

While our enrichment was not perfect (three cells from the HEK293T-targeted sample that aligned to the mouse genome and the one cell from the NIH/3T3-targeted sample that aligned to a human genome), we hypothesize that this is due to background noise in non-photoactivated cells’ fluorescence levels: 8.72 and 15.32% of the HEK293T- and NIH/3T3-targeted cells were fluorescein isothiocyanate (FITC) channel positive, respectively, and the non-photoactivated control had a 0.19% false positive rate in the FITC channel; thus, in the two enriched datasets, we would expect roughly 1 error, in close agreement with the 3 and 1 observed. More stringent gating could thus improve purity but may reduce yield.

Collectively, these data demonstrate that the photoactivated calcein NVOC fluorescence signal persists through dissociation and can be detected by flow cytometry, demonstrating SPACECAT’s utility for tagging and extracting cells.

### Cell tagging of budding crypts in human intestinal organoids confirms stem-like program

**Fig. 2:**
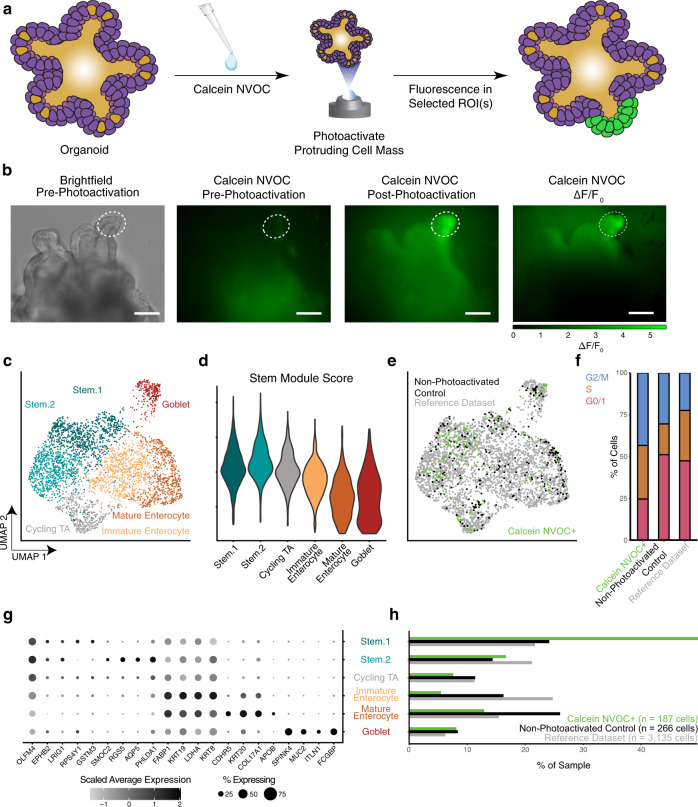
Application of SPACECAT for physical isolation of stem cell niches from human small intestinal organoid models. **a** Schematic of organoid crypt selection with the SPACECAT protocol. Organoid graphic adapted with permission from ref. ^67^. **b** Representative images of cultured organoids and example selected regions for photoactivation and selection, as well as changes in fluorescence normalized to baseline fluorescence. Dashed line: photoactivated region boundary; scale bar = 100 μm. *n* = ~70 regions from the periphery of ~20 organoids, targeting 10–20 regions per protrusion. **c** UMAP of 3,588 single cells in total, from non-photoactivated control organoids (*n* = 266 cells), sorted calcein NVOC+ cells from photoactivated organoids (*n* = 187 cells), and an internal reference dataset of organoids derived from the same patient and cultured identically (n = 3135 cells); cells colored by annotated cell type. **d** Scoring of cell types for expression of a module of stem-associated genes (LGR5, AXIN2, FZD7, OLFM4, LRIG1), following the approach of Tirosh et al.^68^. **e** UMAP of organoid dataset, colored by experimental condition (non-photoactivated controls: black; calcein NVOC+: green; reference dataset: gray). **f** Cell cycle stage by isolation condition as determined by gene module scoring (Tirosh et al.^68^), *p* value < 0.001 when comparing Calcein NVOC+ cells to either other cell population by Chi-square test (Calcein NVOC+: 187 cells, green; non-photoactivated control: 266 cells, black; reference dataset: 3,135 cells, gray; *p* value = 7.404 × 10^−8^ for calcein NVOC+ vs. non-photoactivated control, *p* value = 3.436 × 10^−12^ for calcein NVOC+ vs. reference). **g** Selected differentially expressed genes marking each cell type. Circle size denotes percentage of each cluster expressing a given gene, circle color denotes relative digital gene expression compared to other clusters. **h** Cluster membership by experimental condition, *p* value < 0.001 when comparing calcein NVOC+ cells to either other cell population by Chi-square test (*p* value = 3.587 × 10^−8^ for green vs. black, *p* value < 2.2 × 10^−16^ for green vs. gray). Source data are available as a Source data file.

While spatial -omics has, in some instances, been enabled with genetic transformations, many samples are not easily amenable to genetic manipulation^4^. To test SPACECAT’s capacity to tag specific spatial features within a complex 3D biological structure in a system where genetic transformation is difficult, we applied SPACECAT to enrich for known morphological features within human intestinal organoids^22^. Human organoids are highly structured and possess significant cellular heterogeneity, with crypt-like protrusions known to be enriched for highly proliferative, stem-like populations. As stem cells cycle, cells migrate from the crypt to the villus and terminally differentiate into other, mature cell types of the intestinal epithelium^23,24^. To isolate cells from crypt regions, we photoactivated ~70 protrusions from the periphery of ~20 organoids, targeting 10–20 cells per protrusion. This produced fluorescence signals that were spatially specific and did not include cells in core or villi regions of the organoids (Fig. 2a, b). Further, the photoactivation produced consistent fluorescence changes throughout the expected depth of targeted protrusions, supporting the ability to use epifluorescence to target cells deeper than the biological structure’s outermost layer (~30 µm; Supplementary Fig. 8a, b). Organoids were dissociated, and populations of calcein NVOC+ cells and background (i.e., non-photoactivated) cells were isolated by FACS. These cells were processed in parallel for scRNA-seq using the Seq-Well platform^25–27^, yielding 187 and 266 calcein NVOC+ and calcein NVOC− cells, respectively (Supplementary Fig. 8c). The recovered 187 cells represent a reasonable proportion of photoactivated cells (~18% of expected photoactivated cells assuming 15 cells/region), given expected losses through dissociation, flow sorting, and library preparation processes. For increased cell counts and additional resolution in identifying cell types, the dataset generated here was combined with a larger reference dataset of organoids derived from the same donor and cultured identically (1,541 and 1,594 cells across two sequencing runs).

Clustering revealed the expected continuum of cell states in an intestinal organoid, from stem populations to cycling transit-amplifying cells, immature enterocytes, and mature enterocytes, as well as a population of goblet cells (Fig. 2c, d, Supplementary Data 1). Critically, SPACECAT enabled us to enrich for cells from the first of two stem cell clusters, which was characterized by stem marker genes like *OLFM4* and *LRIG1* (Fig. 2e–h). Using previously described gene lists^28^, we scored each cell for its position in the cell cycle and categorized the cells into various stages (G0/1, S, and G2/M; Fig. 2f). We found that a significantly larger proportion of calcein NVOC+ cells from the protruding crypt regions had entered the cell cycle (S or G2/M) compared to non-photoactivated cells (Fig. 2f; *p* value < 0.001, Chi-squared test). Calcein NVOC+ cells were also significantly enriched for stem-like populations (e.g., expression of stem cell markers like *OLFM4* and *LRIG1*; markers like *GSTM3* known to be crypt-localized)^29^, while the bulk organoid exhibited higher proportions of cycling transit-amplifying cells, immature enterocytes, and mature enterocytes. (Fig. 2g, h; *p* value < 0.001, Chi-squared test). These results match well with prior literature on cell profiles derived from intestinal organoid crypts, which have previously been shown to be the location of actively cycling stem cell populations capable of multipotent differentiation into the diverse intestinal epithelium cell types^30–33^.

Importantly, this dataset also allowed us to evaluate potential negative effects of photoactivation on cellular phenotypes. We compared gene expression patterns between photoactivated cells in the Stem.1 cell cluster (the cluster most enriched by photoactivation) against non-photoactivated control cells in the same cluster from control organoids processed on the same day. Common quality metrics like percentage of mitochondrial unique molecular identifiers (UMIs) were not higher in photoactivated stem cells (*p* value > 0.9, Mann–Whitney *U* test), and genes commonly associated with low-quality cell health (e.g., *NEAT1*, *MALAT1*) were expressed at lower levels in photoactivated stem cells than in stem cells from non-photoactivated controls (*p* value < 0.05, Mann–Whitney *U* test with Bonferroni correction; absolute value of Cohen’s *D* >1 for *NEAT1* and *MALAT1*)^34^. More broadly, 141 genes (out of 25,441) were differentially expressed in stem cells between the photoactivation conditions (*p* < 0.05, Mann–Whitney *U* test with Bonferroni correction; Supplementary Data 2). However, they did not exhibit consistent shared features or functions: gene set enrichment analysis returned no statistically significant associations with lists in MSigDB’s Hallmark collection (50 gene sets in total), no associations with lists in the Gene Ontology Human Phenotype Ontology collection (4,494 gene sets in total), and 2 associations with lists in the Gene Ontology Molecular Function collection (RNA Binding, Structural Molecule Activity; 1,697 gene sets in total). Combined with lack of damage-associated changes in quality-control phenotypes of photoactivated cells, these results indicate that the brief exposure to photoactivation illumination used in SPACECAT tagging does not negatively impact cell health or relevant phenotypes (e.g., intestinal function, stemness). Thus, our findings align with current models of intestinal organoid structure, demonstrating that SPACECAT enables selecting, labeling, and enriching of cells from user-defined regions within human organoids without significant impacts on cellular phenotypes.

### Calcein NVOC is compatible with diverse tissues

**Fig. 3:**
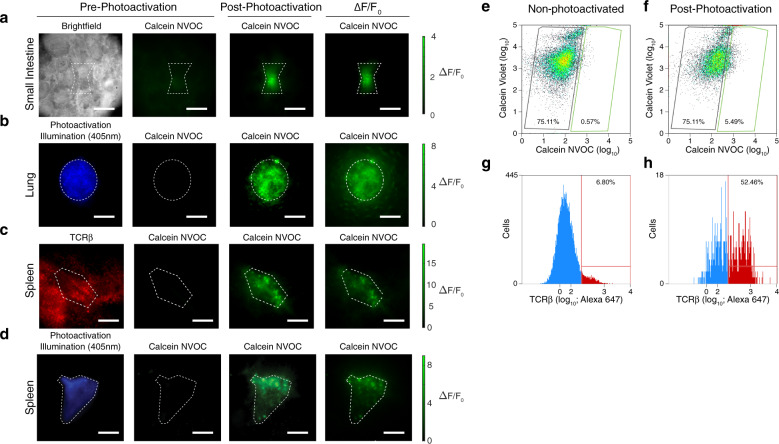
SPACECAT enables tagging of precise regions in live tissue sections. **a**–**d** Various mouse tissue samples were sectioned live, stained with calcein NVOC, and photoactivated in arbitrary regions (within white dashed boundaries). **a** Small intestine (*n* = 3 independent fields of view; scale bar = 50 μm), **b** Lung (*n* = 3 independent fields of view; scale bar = 50 μm). **c**, **d** Representative images of mouse spleen (*n* = 3 independent fields of view); T cell-rich areas were selected for photo-tagging (within dashed white boundary). Left: TCRβ chain antibody (red, **c**) or photoactivation illumination (blue, **d**); left middle: calcein NVOC prior to photoactivation; right middle: calcein NVOC after photoactivation; right: calcein NVOC Δ*F*/*F*_0_. Scale bar = 50 μm. **e**, **f** Flow cytometry of calcein NVOC (*x*-axis, marker of viability and photoactivation) vs. calcein violet (*y*-axis, marker of viability) in non-photoactivated (**e**) and photoactivated (**f**) splenic slices of T cell-rich areas. **g**, **h** Abundance of TCRβ-positive cells among all splenic cells (**g**) and photoactivated cells (**h**) from photoactivated splenic slices.

To demonstrate the versatility of SPACECAT in complex tissue microenvironments, we photoactivated regions in live tissue slices of mouse small intestine, lung, spleen, and brain (Fig. 3a–d and Supplementary Fig. 9a). Despite the range of cell types and tissue structures (e.g., extracellular matrix types and composition) within these tissues, photoactivation of calcein NVOC provided spatially localized fluorescence signals that were significant above background. Notably, we did not adapt the protocol between distinct tissue types, demonstrating wide applicability and compatibility.

To test the SPACECAT cell tagging protocol within a heterogeneous tissue and retention of calcein NVOC fluorescence signal through tissue dissociation, we photoactivated T cell-rich regions found in close proximity to vasculature within the spleen, likely representing periarteriolar lymphoid sheaths (Fig. 3c and Supplementary Fig. 9a)^35^. Such T cell-rich zones (white pulp) are important known sites of antigen presentation by activated dendritic cells and initiation of adaptive immune responses. Yet, beyond histological and imaging evaluations, the proportion of T cells in periarteriolar lymphoid sheaths is not widely established, due to difficulties with such spatially resolved measurements. A study relying on differential susceptibility of red vs. white pulp to collagenase reported that the periarteriolar lymphoid sheath contains ~51% T cells^36^. Here we applied the SPACECAT protocol as an independent, spatially resolved measurement of T cell proportion within T cell-rich zones of the spleen.

Photoactivation of T cell-rich perivascular regions produced calcein NVOC fluorescent cell populations that were not present before photoactivation, as assessed by both imaging and flow cytometry (Fig. 3c–f and Supplementary Fig. 9a, b). When analyzed further by flow cytometry, the fluorescent cell population contained 52% T cells, in close agreement with the 51% value previously reported (*p* < 0.0001 for enrichment of T cells in photoactivated vs. non-photoactivated spleen, Fisher’s exact test, Fig. 3g, h). Furthermore, while not directly tested in this experiment, we began photoactivation of spleen sections 7 h before signal was measured by flow cytometry, supporting our in vitro time course demonstration of extended fluorescence signal stability (Fig. 1c and Supplementary Fig. 6c, d). Targeting microanatomical features for imaging or FACS with a fluorescent antibody supports robust spatial tagging in tissue contexts and represents an alternative endpoint to the single-cell transcriptomics demonstrated in the organoid model.

### User-directed cell tagging uncovers spatial heterogeneity in tumor-infiltrating myeloid cells

**Fig. 4:**
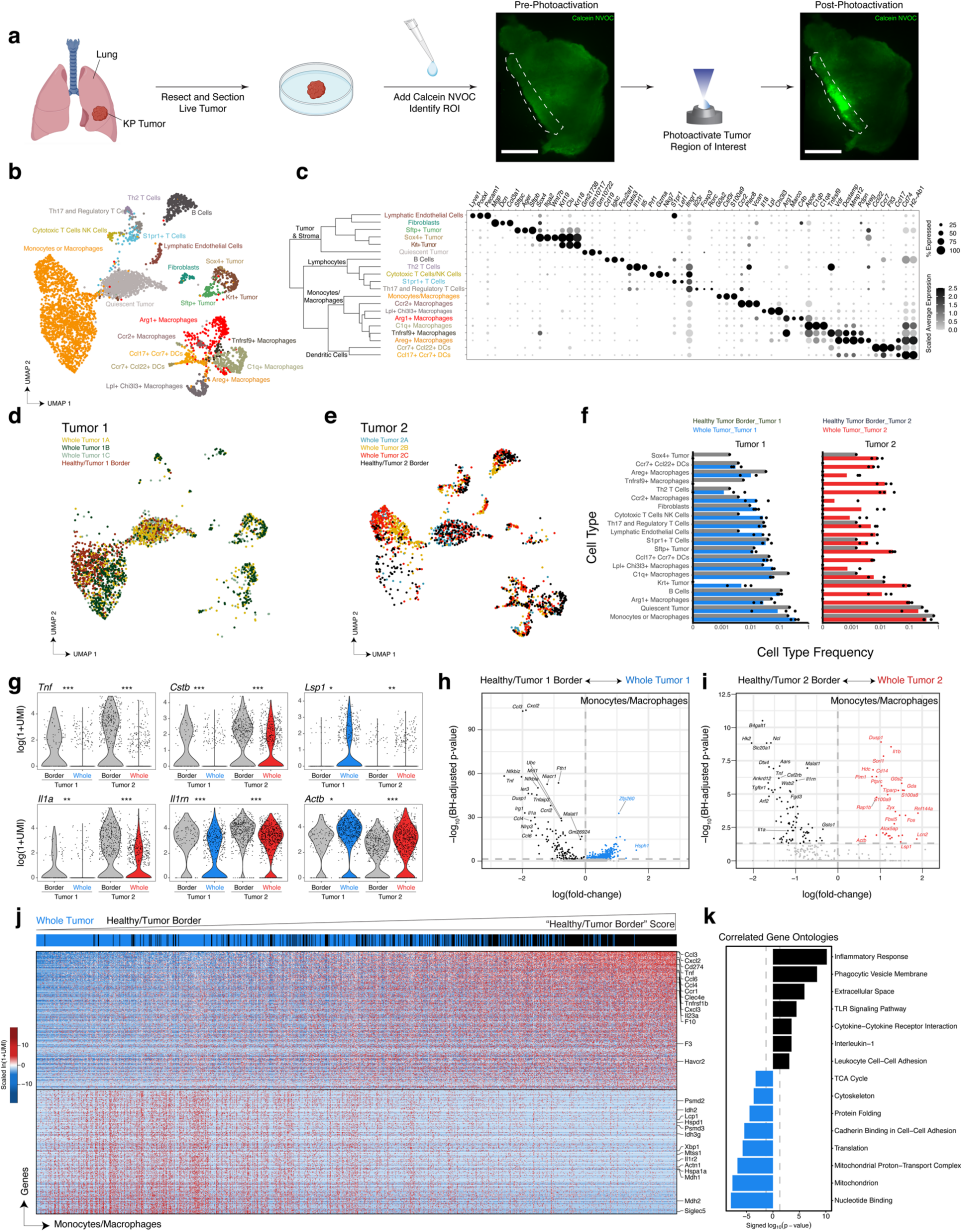
Applying SPACECAT to a KP autochthonous lung tumors uncovers spatial heterogeneity of immune infiltrate. **a** Experimental schematic and representative stitched images of KP lung tumors slices in FITC fluorescence channel before photoactivation (left) and after photoactivation of regions within the white dashed boundary (right). Scale bar = 500 μm. Created using BioRender.com (2021). **b** UMAP embedding and Louvain clusters of 4343 single cells, colored by annotated cell type and state. From Tumor 1, 1634 were from the non-photoactivated, total population and 686 were from the Healthy/Tumor Border. From Tumor 2, 1489 were from the non-photoactivated population and 534 from the Healthy/Tumor Border. **c** Hierarchical clustering of cell types and dot plot of top differentially expressed genes between each cluster and all other cells. All genes significant by Bonferroni-adjusted *p* value <0.001. Dot color represents average expression per cluster; dot size represents percentage of expression per cluster. **d** UMAP embedding with points corresponding to cells only in Tumor 1. Points colored by sort condition; yellow, green, and gray represent triplicate Seq-Well arrays from the Whole Tumor. Red points represent single cells photoactivated and sorted from the Healthy/Tumor Border. **e** UMAP embedding with points corresponding to cells only in Tumor 2. Points colored by sort condition; blue, yellow, and red represent triplicate Seq-Well arrays from the Whole Tumor. Black points represent single cells photoactivated and sorted from the Healthy/Tumor Border. **f** Relative cell-type compositions colored by region and tumor. Individual dots represent proportions in the triplicate technical replicates of three Seq-Well arrays from the Whole Tumor for each tumor (blue for Tumor 1, red for Tumor 2) or single Seq-Well array from the Healthy Tumor Border (black). *X*-axis presented in log scale, and length of bar indicates mean proportion across triplicate technical replicates. **g** Expression of individual genes among Monocyte or Macrophage cells by tumor region in Tumor 1 and 2 (Healthy/Tumor Border vs. Whole Tumor). Two-sided likelihood ratio test for single-cell expression with Bonferroni-adjusted *p* value ***<0.001, **<0.01, *<0.05 (*n* genes = 27,923); *p* values for Tumor 1 and Tumor 2, respectively for: *Tnf*: 5.103442 × 10^−59^ and 1.230068 × 10^−^^07^, *Cstb*: 4.442659 × 10^−^^06^ and 1.851645 × 10^−05^, *Lsp1*: 3.122840 × 10^−03^ and 3.862113 × 10^−02^, *Il1a*: 1.376249 × 10^−30^ and 5.629442 × 10^−03^, *Il1rn*: 2.620537 × 10^−19^ and 9.675296 × 10^−07^, *Actb*: 5.392148 × 10^−12^ and 1.491589 × 10^−02^. **h**, **i** Volcano plots of differentially expressed genes between Monocyte and Macrophage cells by tumor region; dashed lines correspond to Bonferroni-adjusted *p* value = 0.05. **h** Healthy/Tumor 1 Border (*n* = 480 cells) vs. Whole Tumor 1 (*n* = 674 cells); blue: higher relative expression in Whole Tumor 1, black: higher relative expression in Healthy/Tumor 1 Border; *n* genes = 27,923. **i** Healthy/Tumor 2 Border (*n* = 104 cells) vs. Whole Tumor 2 (*n* = 527 cells); red: higher relative expression in Whole Tumor 2, black: higher relative expression in Healthy/Tumor 2 Border; *n* genes = 27,923. **j** Heatmap of 321 genes negatively and 360 genes positively correlated with Healthy/Tumor Border Score among Tumor 1 Monocyte/Macrophage cells (blue: lower relative expression, red: higher relative expression). Cells are ordered by expression of Healthy/Tumor Border Score (color bar represents original location of each cell: blue, Whole Tumor; black, Healthy/Tumor Border). All genes significant by *p* value < 0.05. **k** *p* Values of significantly enriched gene ontologies among genes positively correlated with Healthy/Tumor Border Score (black bars) and genes negatively correlated with Healthy/Tumor Border Score (blue bars). Dashed lines correspond to *p* value = 0.05. Source data are available as a Source data file.

Finally, we applied SPACECAT to a genetically engineered mouse model of lung adenocarcinoma that phenotypically recapitulates key aspects of human disease. In the Kras-p53 (*Kras*^LSL-G12D/+^, *p53*^fl/fl^; KP) model^37^, intratracheal administration of adenovirus expressing Cre recombinase induces oncogenic *Kras* expression with simultaneous deletion of *p53*, leading to transformation of alveolar type II cells in the context of the healthy lung stroma and natural immune response. To test SPACECAT in the KP model, we dissected whole tumors 16 weeks post initiation from the lungs of two mice (one male, one female) and interrogated the tumor periphery by exposing intact tumor sections to calcein NVOC, photoactivating desired regions, sorting cells by calcein NVOC fluorescence, and generating scRNA-seq libraries using Seq-Well (Fig. 4a and Supplementary Fig. 9c–f).

After preprocessing and filtering for high-quality cells, we recovered a total of 4343 single cells and 27,923 unique genes from two tumors (Tumor 1: 686 cells from the photoactivated Healthy/Tumor Border region and 1634 cells from the Whole Tumor; Tumor 2: 534 cells from the photoactivated Healthy/Tumor Border and 1489 from the Whole Tumor; Supplementary Data 3). For cell-type identification, we reduced this high-dimensional data into a lower-dimensional manifold using principal component analysis (PCA) over variable genes, clustered cells using a mutual *k* nearest-neighbor graph, and visualized these clusters on a Uniform Manifold Approximation and Projection (UMAP) embedding (Fig. 4b)^38^. This approach uncovered 20 distinct cell types representing multiple known lymphoid, myeloid, and stromal populations associated with the KP tumor model, including 4 distinct subpopulations of tumor cells, termed *Sox4+* Tumor, *Krt+* Tumor (defined by upregulation of multiple keratin genes, including *Krt19*, *Krt18*, *Krt7*), *Sftp+* Tumor (defined by upregulation of surfactant genes, including *Sftpb*, *Sftpc, Sftpd*), and a Quiescent Tumor cell type (Fig. 4b, c, Supplementary Data 4). Note that, due to the method of dissection, we hypothesize these are predominately tumor cells though there may be healthy epithelial cells within these clusters. Further, we recovered nine populations of monocytes, macrophages, and dendritic cells represented in both tumors. We termed the largest subpopulation of myeloid cells “Monocytes or Macrophages,” defined by expression of well-described markers of monocytes and activated macrophages, including *Csf3r*, *G0s2*, *Cd14*, and *Fcgr3*, as well as multiple inflammatory cytokines, including *Tnf*, *Ccl4*, *Cxcl3*, *Il1b*, *Il1a*, and *Ccl3*. The “Monocytes or Macrophages” cell cluster is distinct from the six other populations of macrophages, which each express a more restricted and mutually exclusive set of functional genes, suggesting greater differentiation and specialization (Supplementary Data 4). Note that, while we did not identify a distinct neutrophil population, these cells may reside within the various monocyte and macrophage clusters.

All cell types were recovered from both Tumor 1 and Tumor 2, however, at different relative abundances (Fig. 4d–f). Notably, in Tumor 2, we recovered a much higher proportion of B cells, T cells, and dendritic cells, potentially indicating either a more immune-permissive environment within Tumor 2, or that we may have captured a tumor-associated tertiary lymphoid structure. To control for tumor-intrinsic heterogeneity, we compared the abundance of cell types between calcein NVOC+ Healthy/Tumor Border sorted cells and cells from the non-photoactivated, Whole Tumor arrays in each tumor separately (Fig. 4f). The Monocytes or Macrophages population was the only cell type with significant differences in abundance between the Healthy/Tumor Border-derived cells and the Whole Tumor across both tumors, albeit in different directions (Benjamini–Hochberg-adjusted *p* value < 0.001 by Fisher’s exact test, expanded and decreased in the Healthy/Tumor border samples in Tumor 1 and Tumor 2, respectively; Fig. 4f). In addition, we observed a significant expansion of *C1q*+ Macrophages in the Tumor/Healthy Border of Tumor 2 (Benjamini–Hochberg-adjusted *p* value < 0.001 by Fisher’s exact test)—this cell type was defined by elevated expression of complement components (*C1qa*, *C1qb*, and *C1qc*), cathepsins (*Ctsh*, *Ctsc*, and *Ctss*), and *Cx3cr1*, which is involved with macrophage migration to inflamed sites. Conversely, in the Tumor 1 Healthy/Tumor Border sample, the majority of the cells were restricted to the Quiescent Tumor or Macrophage/Monocyte cell types with significant differences observed in *Arg1*+ Macrophages, *Tnfrsf9*+ Macrophages, *Krt*+ Tumor cells, *Sftp*+ Tumor cells, and Th2 T cells between the Healthy/Tumor Border and Whole Tumor (Benjamini–Hochberg-adjusted *p* value < 0.001 by Fisher’s exact test). Certain genes involved in key immunological processes were reproducibly expressed at either higher or lower levels in the Healthy/Tumor Border-derived monocytes and macrophages as compared to their Whole Tumor counterparts across this *N* = 2 study (Fig. 4g). Together, this data supports profound inter-tumor, as well as intra-tumor spatial heterogeneity among tumor subtypes and infiltrating lymphocytes within the KP tumor model.

To complement the capabilities of SPACECAT’s experimental spatial tagging, we developed and implemented a computational framework for analyses building on the results of cell-type classification, differential frequency, and differential expression comparisons. To infer smooth gradients along the axis between tagged regions, we scored cells on the expression of differentially expressed genes between tagged regions and identified genes that correlated with that gene expression signature. Given the observed significant changes in the relative abundance of Monocyte/Macrophage between the Healthy/Tumor border and Whole Tumor samples from both tumors, we chose to focus on these cell types as an example of investigations enabled by SPACECAT. From Tumor 1, we observed 117 genes downregulated and 372 genes upregulated in the Whole Tumor compared to the Healthy/Tumor Border (Fig. 4h and Supplementary Data 4); from Tumor 2, we observed 31 downregulated and 80 upregulated genes in the same comparison (Fig. 4i and Supplementary Data 4). We then scored and ranked all cells from Tumor 1 belonging to this cluster by their expression of the 117 genes with increased expression among Monocytes/Macrophages at the Healthy/Tumor Border in Tumor 1. There were 321 genes negatively correlated and 360 genes positively correlated with the Healthy/Tumor Border score; ordering cells by their Healthy/Tumor Border score demonstrated smooth and opposing gradients in gene expression, pointing toward SPACECAT’s ability to uncover potentially important, yet nuanced patterns of spatial variation within complex microenvironments (Fig. 4j). Gene ontology enrichment revealed the cellular pathways associated with this ranking inclusive of several enrichments of potential relevance to the spatial organization of the tumor microenvironment (Fig. 4k and Supplementary Data 4). While this study is underpowered to make definite conclusions about relevant features of the tumor microenvironment in this model (see Supplementary Note 1 for a discussion of power to detect rare cells), here we demonstrate that SPACECAT enables lines of spatial inquiry in ex vivo samples from diverse biological settings and specifically adds spatial context to recent epigenetic and longitudinal transcriptomic investigations within the KP tumor model^39,40^.

## Discussion

SPACECAT provides a broadly applicable method for fluorescently staining cells in arbitrary ROIs, tracking live cells over extended periods of time, and linking information about a cell’s spatial location or dynamics to myriad other measurements (e.g., single-cell -omic measurements, flow cytometry). Using in vitro cell cultures, we demonstrated cell tagging with spatially specific and temporally stable fluorescent labeling. In 3D human organoids, we confirmed known biological structures of stem cell-enriched, mitotic crypt niches, and in spleen tissue sections, we targeted T cell-rich zones to provide a quantitative measurement of T cell fraction independent of prior methods. To demonstrate SPACECAT’s ability to enable discovery of biological phenotypes, we further applied our technique to cross-sections of an autochthonous lung tumor mouse model and uncovered spatially enriched rare subsets of monocytes exhibiting transcriptional programs associated with differentiation and immune recruitment, suggesting promising directions for further inquiries and biological validation focused on the spatial structure underlying heterogeneity in cancer–immune crosstalk.

In addition to applications across diverse model systems, these trials highlight SPACECAT’s strength for spatially informed assays. The temporal stability of the encoded fluorescence signals enables tagging and tracking of cellular dynamics over several hours, important for examining biological function (e.g., in a stage-top incubator to preserve sample viability throughout tagging, time course imaging, isolation, and downstream assays). Our longest demonstration of temporal stability was 16 h in a dish of rapidly dividing cells from a cancer cell line, but further tracking is possible (note that calcein is a commonly used viability dye across multiple organs, including for labeling cells for times as long as days)^12–14,17,18^. The durability of SPACECAT’s fluorescent signals will depend on sample characteristics (e.g., longer signal duration in slowly dividing cells, shorter duration in metabolically active cells). Spatially, SPACECAT’s ability to fluorescently stain regions as small as individual cells enables tagging of complex regions, important for labeling the geometries of biological structures (with subsequent isolation dependent on the set-up and hardware of downstream flow sorting).

Our organoid trials also point toward possibilities for fluorescent tagging into the depth of a biological structure. As part of organoids’ experimental preparation, they retained a surrounding shell of Matrigel, providing an indication on the ability of active illumination to penetrate into complex biological environments (e.g., extracellular matrix mimicked by Matrigel). More sophisticated photoactivation approaches than the digital micromirror device used here (e.g., two-photon microscopy) or optimized protocols (e.g., increased photoactivation exposure lengths, pulsed photoactivation protocols) have the potential to increase the penetrance depth or more specifically define axial staining. The use of a standard epifluorescence microscope provides a conservative indication on SPACECAT’s capabilities. We present the trials here to serve as key biological contexts where SPACECAT enabled previously difficult, spatially resolved assays with in vitro cell lines, organoids, and tissue sections.

For future applications of SPACECAT, the demonstrations across model systems exemplify various approaches for determining ROIs in biological samples. Researchers could define SPACECAT ROIs based on prior expectations of spatial variations in gene expression associated with biochemical availability (e.g., edge–core differences in a tumor as demonstrated in this manuscript). As a related example, researchers could rely on knowledge of tissue structures, such as separately profiling regions along the crypt-to-villus progression in intestinal tissue sections (building on our demonstrations in this manuscript isolating cells from organoid crypts). As a third option, we harnessed cell-type-specific fluorescent antibodies to target the periarteriolar lymphoid sheath surrounding arterioles in the spleen. Importantly for spatial tagging given limited fluorescence channels, we demonstrate multiplexing capability, encoding 6 distinct spatial regions with 3 fluorescence channels in Fig. 1e (5 regions with distinct fluorescence intensity combinations + 1 background null region). Spatial heterogeneity could be further explored by investigating adjacent slices of tissue section for different features. By leveraging a flexible number of fluorescence channels for SPACECAT tagging or labels/antibodies, along with prior hypotheses and known morphologies, the SPACECAT protocol can be adopted for a variety of spatial investigations.

In this work, we have leveraged imaging, flow cytometry, and scRNA-seq as final readouts after cell tagging using the SPACECAT protocol. Importantly, this approach yields live cells and allows for significant experimental flexibility. Cells can either remain in situ for dynamic tracking and staining or can be removed from that environment to be evaluated by biophysical assays, functional characterization, genomics methods, or potentially a combination of these assays^41,42^. These features make SPACECAT a promising addition to the spatial -omics toolbox, especially as a complement to techniques that require lethal endpoint preparations of biological samples. For instance, techniques like in situ sequencing^7^, STARMAP^43^, or fluorescence in situ hybridization^44,45^ enable terminal single-cell genomic profiling across a tissue section, at the expense of potentially complex chemistries and/or sample processing protocols and imaging hardware set-ups. Slide-seq^46^ or high-definition spatial transcriptomics^47^, meanwhile, provide spatial information in an experimental format comparable to typical single-cell sequencing assays, with potential tradeoffs related to transcript capture efficiency and appropriate interpretation of single-bead measurements (as opposed to single-cell measurements). Ultimately, all of these techniques (and others not listed here but reviewed elsewhere^48,49^) deliver complementary capabilities with their own particular use cases and could even be coupled with SPACECAT to link cellular dynamics to end-state phenotype^49^. We envision SPACECAT serving as a strong small molecule addition to the set of recently developed methods for spatially informed assays that leverage photocaged olignucleotides^50^ and nanobodies^51^. With a minimally perturbative, temporally stable, and spatially precise fluorescent-tagging signal, the SPACECAT protocol can be incorporated into existing sample workflows to increase information produced from an experiment, requiring only the addition of specific dyes described here and elsewhere and an active UV illumination source^19^. By exposing a sample to different dyes, separated by washing and photoactivation steps, this protocol can yield tagged cells from multiple regions to be compared and contrasted. Importantly, the number of possible regions scales multiplicatively with the number of dyes at varying photoactivation thresholds, enabling multiple neighborhoods to be stained from a limited set of encoding dyes.

By enhancing endpoint readouts—whether they be cell growth rate, cytokine secretion, genomics, etc.—with positional information (e.g., interfaces between diseased tissue and normal parenchyma, proximity to lymphatics or a mucosal surface) or by tracking cells of interest through space and time without genetic modifications, SPACECAT has the potential to further our understanding of connections between microscale cellular characteristics, mesoscale intercellular architecture, and macroscale biological function.

## Methods

### Calcein NVOC synthesis

#### 4’,5’-Dimethyl-3’6’-diacetoxyspiro[isobenzofuran-1(3H),9’-[9H]xanthen-3-one (**1**)

Phthalic anhydride (16.7 g, 0.113 mol) and resorcinol (24.9 g, 0.206 mol) were charged in an oven-dried 250 mL round-bottomed flask under nitrogen and slowly heated with the use of a sand bath (thermocouple probe in bath = 170 °C) until the solids were completely melted. Anhydrous zinc chloride (15.0 g, 0.110 mol, weighed in an argon-filled glove box) was added portion-wise against positive nitrogen pressure as the sand bath temperature was slowly raised to 230 °C. After approximately 30 min (as indicated by the literature), no solidification had occurred, so the temperature was further raised to 250 °C, at which point the reaction began releasing water vapor.

A vent needle was added, and the reaction was left at a thermocouple temperature of 250 °C until it had solidified as a dark red solid. The flask was removed from the sand bath and was allowed to cool in air for 1 h. The solid was then removed and pulverized with a mortar and pestle.

The resulting powder was suspended in 6 N hydrochloric acid (150 mL) and heated to reflux for 30 min. The resulting solid product was collected by vacuum filtration, washed with water, and air-dried with flowing compressed air and continual vacuum pressure. The solid obtained was carried on crude into the next step.

This crude solid was suspended in acetic anhydride (300 mL) in a round-bottom flask under nitrogen atmosphere and heated to a bath temperature of 100 °C. Once it reached that temperature, pyridine (20 mL) was slowly added down from the top of the attached reflux condenser, and the reaction was left to stir overnight. A shorter reaction time could likely be applied if desired, as the analogous reaction with benzoic anhydride is reported to be complete in 2.5 h. In the morning, thin-layer chromatographic (TLC) analysis indicated complete consumption of the starting material (as judged against a small sample of crude solid reserved from the first step). The reaction was allowed to cool and was then poured onto approximately 300 g of ice to induce precipitation.

After the ice had melted, the resulting solid was collected by filtration and washed with water. Recrystallization from acetone yielded 24.2 g (50% yield) of a creamy solid that produced a colorless solution upon dissolution.

^1^H nuclear magnetic resonance (NMR; 401 MHz, chloroform-*d*) *δ* 8.17–7.91 (m, 1H), 7.66 (dtd, *J* = 21.9, 7.4, 1.2 Hz, 2H), 7.22 (d, *J* = 7.5 Hz, 1H), 6.78 (d, *J* = 8.7 Hz, 2H), 6.68 (dd, *J* = 8.7, 0.7 Hz, 2H), 2.36 (s, 6H), 2.35 (s, 6H).

^13^C NMR (150.9 MHz, chloroform-*d*) *δ* 168.8, 152.8, 150.4, 150.2, 135.1, 129.9, 126.4, 125.5, 125.1, 124.2, 119.1, 117.7, 116.4, 82.6, 20.8, 9.5. High-resolution mass spectrometry (electrospray ionization) HRMS (ESI) calculated for C_26_H_20_O_7_ [M + H]^+^: 445.12873. Found 445.12898 (Supplementary Fig. 2).

#### 4’,5’-Bis-(bromomethyl)-3’6’-diacetoxyspiro[isobenzofuran-1(3H),9’-[9H]xanthen-3-one (**2**)

This material was synthesized by adapting a procedure from a Lippard patent^52^. The methyl fluorescein derivative prepared above (3.91 g, 8.80 mmol) and 1,3-dibromo-5,5-dimethylhydantoin (3.87 g, 13.5 mmol) were charged in a 1-L flask with a Schlenk adapter attached. Chlorobenzene (550 mL) was added, and the solution was degassed by freeze–pump–thaw. 1,1’-azobiscyclohexanecarbonitrile (180 mg, 0.74 mmol) and acetic acid (140 µL) were added against positive nitrogen pressure and the reaction was heated to 40 °C for 72 h, during which time the color changed from nearly clear, to a bromine-related orange, and finally to yellow.

The chlorobenzene solution was transferred into a separatory funnel with the aid of additional toluene and then rinsed with four portions (250 mL each) of water heated to between 50 and 75 °C. The organic layers were dried over MgSO_4_ and concentrated to yield a crude solid, which was recrystallized from 9:1 toluene:EtOH. The resulting solid was collected by vacuum filtration and rinsed with a small amount of cold toluene. The filtrate solution was concentrated and recrystallized a second time from toluene/EtOH to yield a second crop of crystals. Both fractions were independently analyzed by ^1^H NMR before being combined (3.64 g total, 68% yield) for storage and further analysis.

^1^H NMR (401 MHz, chloroform-*d*) *δ* 8.12–7.99 (m, 1H), 7.70 (dtd, *J* = 23.8, 7.4, 1.2 Hz, 2H), 7.32–7.21 (overlapping with CDCl3 signal, 1H), 6.92 (d, *J* = 8.8 Hz, 2H), 6.83 (d, *J* = 8.8 Hz, 2H), 4.82 (s, 4H), 2.42 (s, 6H).

^13^C NMR (150.9 MHz, chloroform-*d*) *δ* 168.7, 168.3, 152.1, 150.3, 149.3, 135.4, 130.3, 128.6, 126.1, 125.4, 124.2, 119.0, 118.8, 116.8, 81.3, 20.9, 20.3. HRMS (ESI) calculated for C_26_H_18_Br_2_O_7_ [M + H]^+^: 602.94771. Found 602.94948 (Supplementary Fig. 3).

#### Iminodiacetic acid bis(2-nitro-4,5-dimethoxy)benzyl ester trifluoroacetate salt (**3**)

2-nitro-4,5-dimethoxybenzyl bromide from commercial suppliers was found to be relatively impure but was upgraded to sufficient purity by dissolving in dichloromethane and filtering through silica (eluted with additional pure dichloromethane) before use. This routinely converted dark brown commercial material into a pale yellow solid after concentration in vacuo.

*N*-Boc-iminodiacetic acid (184 mg, 0.79 mmol), purified 2-nitro-4,5-dimethoxybenzyl bromide (460 mg, 1.67 mmol), and cesium carbonate (542 mg, 1.66 mmol) were charged in an oven-dried 25 mL round-bottomed flask under nitrogen. Dry acetonitrile (4.6 mL) was added, and the solution was left to stir at room temperature for 1 h. TLC analysis at that point indicated slow conversion, and so the reaction was heated to 50 °C overnight (approximately 16 h).

In the morning, TLC showed trace residual bromide (as it was added in slight excess) and one major new UV-active product. The reaction solution was transferred to a separatory funnel and diluted with saturated aqueous sodium carbonate, which was then extracted thrice with dichloromethane. The combined organic extracts were dried over MgSO_4_, filtered, and concentrated *in vacuo*.

To remove excess bromide, the crude product was re-dissolved in a small amount of dichloromethane and loaded onto a silica plug. Elution with pure dichloromethane was performed until all excess bromide had been removed, and then further elution with 10% ethyl acetate in dichloromethane delivered the major new UV-active compound as a single spot.

After concentration *in vacuo*, the resulting intermediate was immediately re-dissolved in dry dichloromethane (4 mL), placed under nitrogen, and cooled to 0 °C. Trifluoroacetic acid (1 mL) was slowly added, and the reaction was left to stir under nitrogen for 4 h. TLC analysis at that point indicated complete consumption of the presumed intermediate. The reaction was allowed to warm to room temperature over the course of 15 min, at which point approximately 15 mL of diethyl ether were added with stirring. This induced immediate precipitation of fine particles. Stirring was stopped and the particles were allowed to flocculate for 10 min before vacuum filtration. The creamy white solid that was obtained was rinsed with a further portion of diethyl ether (approximately 10 mL), before being air dried for 10 min. The creamy white solid was then transferred into a tared 20 mL scintillation vial and further dried under high vacuum to deliver the desired trifluoroacetate salt (458 mg, 91% yield).

^1^H NMR (401 MHz, methanol-*d*_4_) *δ* 7.78 (s, 1H), 7.19 (s, 1H), 5.63 (s, 2H), 4.15 (s, 2H), 3.97 (s, 3H), 3.93 (s, 3H).

^13^C NMR (105.9 MHz, methanol-*d*_4_) *δ* 166.5, 153.7, 149.1, 140.4, 124.7, 111.8, 108.2, 64.6, 55.6. Two carbon peaks are unobserved and are likely found underneath the solvent residual. HRMS (ESI) calculated for [M]^+^ C_22_H_26_N_3_O_12_: 524.15110. Found 524.15164 (Supplementary Fig. 4).

#### Calcein NVOC (**4**)

This material was synthesized by adapting a procedure from the patent literature^53^. A portion of the trifluoroacetate salt generated above was transferred directly into a separatory funnel and suspended in dichloromethane. The dichloromethane was washed twice with an approximately equal volume of 1 M Na_2_CO_3_ solution and once with water. The dichloromethane layer was then dried over MgSO_4_, filtered, and concentrated into a flame-dried, tared 50 mL round-bottomed flask. After concentration *in vacuo* and weighing, it was determined that 303 mg of the free base of iminodiacetic acid bis(2-nitro-4,5-dimethoxy)benzyl ester had been delivered (0.579 mmol).

4’,5’-Bis-(bromomethyl)-3’6’-diacetoxyspiro[isobenzofuran-1(3*H*),9’-[9*H*]xanthen-3-one (135 mg, 0.224 mmol), sodium iodide (70 mg, 0.467 mmol), and 1,8 bis(dimethylamino)naphthalene (97 mg, 0.453 mmol) were added to the round-bottom flask, which was then capped with a septum and purged with flowing nitrogen for 15 min. Dry and degassed acetonitrile was added (6 mL), and the reaction was heated to 75 °C overnight (approximately 18 h).

The reaction was allowed to cool to nearly room temperature and was then filtered through a Kimwipe® pressed into the neck of a standard Pasteur pipette to remove a brightly colored precipitate. Concentration in vacuo yielded a sticky solid that was then suspended in approximately 10 mL dichloromethane, resulting in a yellow solution with white precipitate remaining undissolved. Filtration again through a Kimwipe® by an identical method was followed by rinsing of the precipitated solid with another 2 portions of dichloromethane (~5 mL each). Concentration in vacuo provided a creamy yellow-orange solid. This material was further purified by recrystallization from dichloromethane/ether, where the desired product was the *soluble* component of the mixture. Further small amounts of solid impurities were removed at this stage by a Kimwipe® filtration. Concentration of the dichloromethane/ether solution was followed immediately by another recrystallization from THF/ether, where the desired product was the *soluble* component of the mixture. A final small amount of solid impurities were removed at this stage by a final Kimwipe® filtration.

At this point, TLC analysis (20% EtOAc/CH_2_Cl_2_) showed three UV-active spots: the desired product, the excess unreacted iminodiacetic acid derivative, and a small amount of 1,8-bis(diamino)naphthalene. That same solvent system was used to isolate the desired product by column chromatography. Concentration *in vacuo* yielded a yellow/orange foam. This foam was broken down to a powder with the aid of a metal spatula, and after removing the final traces of solvent with extended time under vacuum (~2 torr, 1 h), a free-flowing orange-yellow powder was obtained (242 mg, 0.163 mmol, 73% yield).

^1^H NMR (400 MHz, acetonitrile-*d*_3_) *δ* 8.03–8.00 (m, 1H), 7.75–7.68 (m, 2H), 7.60 (s, 4H), 7.27–7.20 (m, 1H), 6.95 (s, 4H), 6.84 (d, *J* = 1.6 Hz, 4H), 5.31 (AB quartet, 8H, *J* = 14.4 Hz), 4.28 (s, 4H), 3.85 (s, 12H), 3.80 (s, 12H of 20H shown integration), 3.79 (s, 8H of 20H shown integration), 2.23 (s, 6H).

^13^C NMR (150.9 MHz, acetonitrile-*d*_3_—DEPT QGPSP sequence) *δ* 171.5, 170.2, 169.7, 154.6, 153.8, 153.0, 151.3, 149.3, 140.7, 136.7, 131.3, 128.8, 127.3, 126.8, 126.0, 124.8, 120.2, 119.8, 117.7, 111.6, 109.1, 82.7, 63.8, 57.0, 56.8, 54.5, 47.6, 21.1. HRMS (ESI) calculated for [M + H]^+^: 1487.38507. Found 1487.38703 (Supplementary Fig. 5).

### Cell culture

Murine NIH/3T3 cells (ATCC, CRL-1658) and human HEK293T cells (ATCC, CRL-11268) were cultured in Dulbecco’s modified Eagle’s medium (DMEM) with glutamate and supplemented with 10% fetal bovine serum (FBS) at 37 °C and 5% CO_2_. Cells were passaged every 2–3 days, as well as on the day of experiments after photoactivation for flow sorting and downstream library preparation. To passage cells, media was aspirated, and the cells were rinsed with phosphate-buffered saline without calcium chloride and magnesium chloride (PBS0; Life Technologies) prior to the addition of Trypsin-LE (Life Technologies). The cells were incubated with Trypsin-LE at 37 °C for 5 min, followed by the quenching with complete media. Cells were pelleted by centrifugation for 5 min at 300 × *g* at 4 °C.

### Organoid culture

All studies were performed under protocols approved by the Massachusetts Institute of Technology (MIT) Committee on the Use of Humans as Experimental Subjects and the Institutional Review Board (IRB) protocols of Massachusetts General Hospital/Partners Healthcare. Small intestinal crypts were isolated from de-identified, IRB-approved human bulk surgical resections. To seed the organoids, the bulk resection was washed with ice-cold Dulbecco’s PBS0 to clear the luminal contents^31,54^. Next 2–4 mm pieces were removed from the epithelial surface with scissors and washed repeatedly by gently pipetting the fragments using a 10-mL pipette until the supernatant was clear. Fragments were rocked on ice with crypt isolation buffer (10 mM EDTA, Life Technologies, in PBS0 supplemented with 500× dilution of Primocin, Invivogen, 10 mM HEPES, Life Technologies, and 2% fetal calf serum, Life Technologies) for 30 min. After isolation buffer was removed, fragments were washed with cold PBS0 by pipetting up and down to release the crypts. Crypt-containing fractions were combined, passed through a 100-μm cell strainer (BD Bioscience), and centrifuged at 50 × *g* for 5 min. The pellet was resuspended in basal culture medium (2 mM GlutaMAX, Thermo Fisher Scientifc, 500× Primocin, and 10 mM HEPES in Advanced DMEM/F12, Life Technologies) and centrifuged at 50 × *g* for 5 min once more to remove single cells. Crypts were then cultured in a Matrigel culture system (described below) in small intestinal crypt medium (50× B27 supplement, Life Technologies, 1 mM *N*-acetyl-L-cysteine, Sigma-Aldrich, in basal culture medium) supplemented with differentiation factors (epidermal growth factor (EGF), Noggin, fibroblast growth factor-2 (FGF-2), insulin-like growth factor 1 (IGF-1), R-spondin 3, Afamin-Wnt3a; concentrations described below) at 37 °C with 5% CO_2_. To plate, crypts were resuspended in basal culture medium at a 1:1 ratio with Corning™ Matrigel™ Membrane Matrix – GFR (Fisher Scientific) and plated at the center of each well of 24-well plates. Following Matrigel polymerization, 500 μL crypt culture medium containing growth factors EGF (50 ng/mL, Life Technologies), Noggin (100 ng/mL, PeproTech), FGF-2 (50 ng/mL, PeproTech), IGF-1 (100 ng/mL, PeproTech), and R-spondin 3 (500 ng/mL, PeproTech) and conditioned media supplement Afamin-Wnt3a (10×, MBL International) was added to each well. ROCK inhibitor Y-27632 (10 μM, R&D Systems) was added for the first 2 days of culture only. Cell culture medium was changed every other day. After 6 days of culture, organoids were passaged and differentiated. Briefly, culture gel and medium were homogenized via mechanical disruption and centrifuged at 300 × *g* for 3 min at 4 °C. Supernatant was removed and the pellet was resuspended in basal culture medium repeatedly until the cloudy Matrigel was almost gone. On the last repeat, the pellet was resuspended in basal culture medium, the number of organoids counted, and centrifuged at 300 × *g* for 3 min at 4 °C. The cell pellet was resuspended in basal culture medium at a 1:1 ratio with Matrigel and plated at the center of each well of 24-well plates (~250 organoids/well). Following Matrigel polymerization, 500 μL crypt culture medium minus growth factor EGF was added to each well. Cell culture medium was changed every other day. Following an additional 6 days of culture, organoids were used in SPACECAT imaging and staining.

### SPACECAT staining

On the day of experiments, aliquots of calcein NVOC (6 M in dimethyl sulfoxide) were diluted 1:100 in polyethylene glycol-200 (PEG-200; Sigma-Aldrich), then in PBS to a final concentration of 1:5000 for photoactivation of organoids and tissue sections or 1:10,000 for photoactivation of in vitro cell lines. PA Janelia Fluor 549 (PA-JF549, Tocris) and PA Janelia Fluor 646 (PA-JF646, Tocris) were reconstituted and diluted according to the manufacturer’s instructions.

Cells were stained for at least 30 min in an incubator at 37 °C and 5% CO_2_. Prior to imaging and photoactivation of in vitro cultures, staining solution was exchanged for phenol-free, FBS-free DMEM, while for organoids and tissue sections, imaging and photoactivation occurred in the staining solution.

### SPACECAT photoactivation of in vitro cell lines

We implemented an Andor Mosaic 3 digital micromirror device on an IX83 Olympus Microscope to achieve the spatially patterned illumination required for user-directed region of interest tagging. The commercially available Mosaic 3 simultaneously illuminates all pixels of all user-created ROIs within a single field of view, enabling rapid processing of many regions as compared with systems that raster laser light using galvanometer-based beam steering (e.g., Rapp Firefly or Andor Micropoint systems). Further, the Mosaic’s resolution is primarily limited by the choice of objective lens, enabling single-cell or even subcellular illumination if desired.

To determine optimal lengths of photoactivation that balance fluorescence signal strength and cellular viability, we photoactivated distinct fields of view with varying near-UV exposure times at ×40 magnification (Supplementary Fig. 6a). We found that exposure to near-UV photoactivation for longer than a total of 4 s caused phototoxicity, as shown through decreases in the fluorescence intensity of calcein NVOC in the 5 s exposure protocol at the 180 min timepoint. However, shorter photoactivation lengths produced similar increases in fluorescence without subsequent effects on viability. As such, we used the combination of 4 s at ×40 magnification as an upper limit on photoactivation exposure, adjusting this limit to account for varying photon fluxes with different magnification lenses. For instance, the field of view with a ×20 lens has a fourfold larger area than does the field of view with a ×40 lens, so that the corresponding photon flux would be fourfold lower. Therefore, to deliver the same number of photons that caused phototoxicity in 4 s at ×40, the upper photoactivation exposure limit with a ×20 lens would be 16 s. However, we frequently used shorter photoactivation exposure lengths than this upper limit, as we could take advantage of comparable signal strengths and increased throughput.

HEK293T cells were seeded in a glass bottom imaging plate (Eppendorf) 2 days prior to photoactivation. On the day of photoactivation, cells were stained with SPACECAT molecules (e.g., calcein NVOC for Fig. 1c; calcein NVOC, PA-JF549, PA-JF646 for Fig. 1e) as described in “SPACECAT staining.” For characterization of SPACECAT’s spatial resolution and temporal stability (Figs. 1e and 3a), cells were also stained with CellTrace Calcein Red-Orange (ThermoFisher) and Hoechst (ThermoFisher) at 1:10^4^ and 1:10^5^ concentrations in Hank’s balance salt Solution (Life Technologies), respectively.

After staining, cells were transferred to a microscope stage top incubator (Tokai Hit) for temperature, humidity, and CO_2_ control throughout photoactivation and imaging. Imaging was conducted on an Olympus IX83 inverted microscope through Olympus UPLSAPO objectives and Semrock bandpass filters, with illumination supplied by a SpectraX Lumencor LED source and images captured on a Hamamatsu ORCA FLASH 4.0LT CMOS camera. Cells were imaged to capture their baseline fluorescence, followed by photoactivation in arbitrary, user-specified regions using 0.5–10 s pulses of 405 nm light controlled by an Andor Mosaic 3 digital micromirror device (Andor Technology, Oxford Instruments), at ×10 magnification. We determined that calcein NVOC was reliably uncaged following 10 s of photoactivation, while PA-JF549 and PA-JF646 dyes could be uncaged after 0.5 s. In this way, we were able to photoactivate five distinct neighborhoods (calcein NVOC alone, PA-JF549 alone, calcein NVOC and PA-JF549, PA-JF549 and PA-JF646, and all three dyes) using just three dyes; additional dyes at each photoactivation threshold would expand the number of possible combinations supralinearly. Subsequent imaging captured cells’ fluorescence levels after photoactivation.

### CellProfiler image analysis of photoactivation specificity and stability

CellProfiler^55^ was used to segment images of SPACECAT photoactivation and quantify the spatial precision and temporal stability of fluorescence signals. Hoechst images were used to identify cells’ nuclei under CellProfiler’s global threshold strategy and robust background thresholding method (lower outlier fraction = 0.05, upper outlier fraction = 0.05, threshold smoothing scale = 10, threshold correction factor = 1.0, suppress local maxima closer than 15 pixels). Cell boundaries were identified under CellProfiler’s Propagation method with Otsu adaptive thresholding of images into two classes (threshold smoothing scale = 10, threshold correction factor = 1, size of adaptive window = 120, regularization factor = 0.01). After segmenting images, each cells’ position within a given image and mean fluorescence over time were calculated, followed by plotting and visualization in MATLAB (MathWorks).

### SPACECAT photoactivation in co-culture environments

HEK293T and NIH/3T3 cells were seeded onto separate pieces of No. 1 coverglass 2 days prior to photoactivation. On the day of photoactivation, the pieces of coverglass were placed abutting each other on Nunc glass bottom dishes (ThermoFisher), and cells were incubated with calcein NVOC as described in “SPACECAT staining.” Depending on the experimental condition, either HEK293T or NIH/3T3 cells within ~2 mm of the species interface region was photoactivated using 10 s pulses of 405 nm light. As a negative control sample, neither species was photoactivated but was exposed to the calcein NVOC molecule. Cells were then lifted from the coverglass as described in “Cell culture”, followed by flow sorting with a Sony SH800Z (gating on calcein NVOC fluorescence levels) into individual wells of a 96-well plate containing 5 μL of Buffer RLT (Qiagen) and 1% β-mercaptoethanol.

### Generation of scRNA-seq libraries using Smart-Seq2

scRNA-seq libraries were generated from single-cell lysates by purifying RNA using AMPure RNA Clean Spri beads (Beckman Coulter) at a 2.2× volume ratio and eluted with 1.9 µL H_2_O, 1 µL oligo-dT Smart-Seq2 RT primer, 1 µL 10 mM dNTPs (NEB), and 0.1 µL RNase inhibitor (Fisher Scientific) at 72 °C for 3 min on a thermal cycler to anneal the 3’ primer to polyadenylated mRNA. Reverse transcription was carried out by adding a mix of 0.65 µL H_2_O, 2 µL Maxima 5X RT Buffer (Fisher Scientific), 2 µL 5 M Betaine (Sigma), 1 µL 10 µM Smart-Seq2 Template Switch Oligo (Supplementary Table 1), 0.9 µL 100 mM MgCl_2_ (Sigma), 0.25 µL RNase inhibitor (Lucigen), and 0.2 µL Maxima RNaseH-minus RT enzyme and using the following protocol: 42 °C for 90 min, followed by 10 cycles of 50 °C for 2 min, 42 °C for 2 min, and followed by inactivation at 70 °C for 15 min^20,21^. Whole-transcriptome amplification was achieved by addition of PCR mix containing: 1 µL H_2_O, 12.5 µL KAPA HiFi HotStart ReadyMix (Kapa Biosystems), and 0.5 µL 10 µM ISPCR primer (Supplementary Table 1) to the reverse transcription product and amplification on a thermal cycler using the following protocol: 98 °C for 3 min, followed by 21 cycles of 98 °C for 15 s, 67 °C for 20 s, 72 °C for 6 min, followed by a final 5 min extension at 72 °C. Libraries were purified using AMPure XP SPRI beads at a volume ratio of 0.8× followed by 0.9×. Library size was assessed using a High-Sensitivity DNA chip (Agilent Bioanalyzer), confirming the expected size distribution of ~1000–2000 bp. Tagmentation reactions were carried out with the Nextera XT DNA Sample Preparation Kit (Illumina) using 250 pg of cDNA per single cell as input, with modified manufacturer’s instructions as described. Libraries were purified twice with AMPure XP SPRI beads at a volume ratio of 0.9×, and size distribution was assessed using a High Sensitivity DNA chip (Agilent Bioanalyzer) and Qubit High-Sensitivity DNA Kit (Invitrogen). Libraries were pooled and sequenced using NextSeq500/550 High Output v2 Kits (75 cycles, Illumina) using 30–30 paired-end sequencing with 8-mer dual indexing.

### Cell line co-culture data processing

Smart-Seq2 libraries cells were sequenced to a depth of 1.3 ± 0.06 million (mean ± standard error of the mean; SEM) reads per cell. Pooled libraries were demultiplexed using bcl2fastq (v2.17.1.14) with default settings and aligned using STAR^56^ to the mouse UCSC genome reference (version mm10) and human UCSC genome reference (version hg19) simultaneously, and a gene expression matrix was generated using RSEM (v1.2.3) in paired-end mode. Cells with <100,000 aligned reads across either genome were eliminated from subsequent analysis. All analysis of gene expression was completed using the normalized RSEM output as transcripts per million.

### SPACECAT isolation of regions from human small intestinal organoids

On the day of photoactivation, organoids were harvested from Matrigel domes and excess Matrigel was removed from organoids through gentle pipetting with a wide-bore P1000 tip. Organoids were briefly spun down at 50 × *g* for 2 min, with supernatant and excess Matrigel carefully removed. Organoids were gently resuspended with wide-bore P1000 tip and placed onto a glass bottom imaging plate (Eppendorf) and stained with calcein NVOC as described in “SPACECAT staining”. Individual protruding crypts were selectively photoactivated with the Mosaic 3 (Andor) as described in “SPACECAT photoactivation of in vitro cell lines”, using 4 s pulses of 405 nm light at ×20 magnification. Organoids were dissociated into a single-cell suspension through brief but vigorous pipetting with a P1000 tip, followed by a 300 × *g*, 5 min pelleting and treatment with Trypsin-LE for 10 min, with vigorous pipetting at 5 and 10 min. Single cells were again pelleted at 300 × *g* for 5 min, and then strained through a 30 µm strainer to exclude clumps. Following dissociation, cells were flow sorted with a Sony SH800Z based on calcein NVOC fluorescence levels.

### SPACECAT isolation of regions from live tissue sections

All studies were performed under animal protocols approved by the Harvard Medical School and MIT Institutional Animal Care and Use Committee. Genetically engineered mice were generated by injecting low passage Kras^Lox-Stop-Lox-G12D/+^; p53^fl/fl^ (KP) embryonic stem cells from the C57BL/6 background into albino C57BL/6 blastocysts^37,39,40^. All animals were housed in static caging (Ancare cages) with hardwood chip bedding, reverse osmosis water, irradiated chow, and a 12/12-h light/dark cycle. Temperature was set to 70 °F with an acceptable range +/−2 degrees with a humidity range of 30–70%. High-degree chimeric mice were identified by coat color and were aged to 12 weeks prior to tumor initiation. Tumors were initiated by intratracheal instillation of 2 × 10^8^ plaque-forming units of Adeno-SPC-Cre (University of Iowa). Sixteen weeks post tumor initiation, mice were euthanized by cervical dislocation and lungs were perfused by cardiac injection with PBS0 prior to removal from the thoracic cavity for fine dissection. Large tumors were identified upon close tissue examination and microdissected with fine surgical tools before placing into RPMI + 10% FBS on ice until further processing.

Tissue samples from the lung, lung tumor, small intestine, and spleen were sliced into ~1 mm sections using an acrylic tissue slicer (Braintree Scientific, Inc.). Brain samples were embedded in 4% low-molecular weight agarose in PBS0 and sectioned live using a vibratome (velocity 1 mm/s, amplitude 0.9 mm, frequency 65 Hz, 150 µm sections) into cold PBS0. After sectioning, tissue was stained with calcein NVOC as described in “SPACECAT staining” for at least 30 min prior to imaging and photoactivation. Brain sections were additionally stained with Hoechst (ThermoFisher) according to the manufacturer’s instructions, while mouse spleen samples were incubated with PE anti-mouse TCR β chain antibody (BioLegend) according to the manufacturer’s instructions (1:200 in RPMI + 10% FBS). Tissue sections were then imaged and photoactivated as described in “SPACECAT photoactivation of in vitro cell lines”, using 12 s pulses of 405 nm light at ×4 magnification.

After photoactivating the spleen and lung tumor sections, samples were dissociated by mechanical and enzymatic methods using the gentleMACS Tissue Dissociation Kit (Miltenyi Biotec). Resultant cell suspensions were stained with calcein violet (ThermoFisher, 1:1000 dilution in PBS0) as an orthogonal indicator of viability. For spleen sections, flow cytometry was performed using a Sony SH800Z quantified cells positive for TCR β chain antibody, while for lung tumor sections, cells were sorted by gating on positive for both calcein violet (viability following dissociation) and calcein NVOC (presence in the ROI). All experiments leveraged calcein NVOC-stained without photoactivation negative control for determining proper gating. Tumor samples were sorted into 500 µL RPMI + 10% FBS and kept on ice for subsequent scRNA-seq.

### Generation of scRNA-seq libraries using Seq-Well

Seq-Well was performed by loading functionalized polydimethylsiloxane nanowell arrays with uniquely barcoded mRNA capture beads (ChemGenes) and suspended in complete media for at least 20 min^25–27^. Fifteen thousand cells or all of what was isolated by FACS was deposited onto the top of each chip and settled into the wells by gravity. The array was gently washed four times with PBS0 and finally with RPMI and sealed using a plasma-functionalized polycarbonate membrane with a pore size of 0.01 µm. Seq-Well arrays were sealed in a dry 37 °C oven for 40 min before being submerged in a lysis buffer containing 5 M guanidium thiocyanate (Sigma), 1 mM EDTA, 1% beta-mercaptoethanol, and 0.05% sarkosyl (Sigma) for 20 min at room temperature. Arrays were washed and then incubated in hybridization buffer containing 2 M NaCl (Thermo Fisher Scientific) with 8% (v/v) PEG-8000 (Sigma) in PBS0 for 40 min at room temperature with gentle rocking. Afterwards, the mRNA capture beads with mRNA hybridized were collected from each Seq-Well array in wash buffer containing 2 M NaCl, 3 mM MgCl_2_, 20 mM Tris-HCl, and 8% (v/v) PEG-8000.

These beads were pelleted at 1000 × *g* for 3 min and resuspended in a master mix for reverse transcription containing: 40 µL H_2_O, 40 µL Maxima 5× RT Buffer (Thermo Fisher Scientific), 80 µL 30% PEG-8000, 20 µL 10 mM dNTPs (Clontech), 5 µL 100 µM 5’ Seq-Well Template Switch Oligo (Supplementary Table 1), 5 µL RNase Inhibitor (Lucigen), and 10 µL Maxima H Minus Reverse Transcriptase (200 µL total) for 30 min at room temperature and overnight at 52 °C with end-over-end rotation. Further washes were performed by pelleting the beads at 1000 × *g* for 1 min and resuspended in the described solution. The beads were then washed once TE with 0.5% sodium dodecyl sulfate (SDS) (TE-SDS, Sigma) and twice with TE with 0.01% Tween-20 (TE-TW, Fisher Scientific) before being resuspended in Exonuclease reaction mix: 170 µL H_2_O, 20 µL 10× ExoI Buffer (NEB), and 10 µL ExoI (NEB; 200 µL total volume) and incubated for 1 h at 37 °C with end-over-end rotation^27^. After exonuclease digestion, beads were washed once with TE-SDS and twice with TE-TW. Beads were then resuspended in 500 µL of 0.1 M NaOH (in H_2_O) and incubated at room temperature for 5 min with end-over-end rotation. Following base denaturation, the beads were washed once with 500 µL TE-TW and once with 500 µL TE and finally resuspended in the Second Strand Synthesis reaction mix: 53 µL H_2_O, 40 µL Maxima 5X RT Buffer, 80 µL 30% PEG-8000, 20 µL 10 mM dNTPs, 5 µL Klenow Fragment (NEB), and 2 µL dN-SMRT Oligo (200 µL total; Supplementary Table 1) to enable random priming for second-strand synthesis during a 1 h incubation at 37 °C. Following second strand synthesis, beads were washed twice with TE-TW, once with TE, once with H_2_O, and then resuspended in 240 µL. In all, 10 µL was distributed into 24 wells of PCR mix per beads sample, each well already contained 25 µL 2× KAPA HiFi Hotstart Readymix, 14.6 µL H_2_O, and 0.4 µL 100 µM ISPCR primer (40 µL total; Supplementary Table 1). PCR was performed using a thermal cycler with the following protocol: 95 °C for 3 min, followed by 4 cycles of 98 °C for 20 s, 65 °C for 45 s, 72 °C for 3 min, followed by 12 cycles of 98 °C for 20 s, 67 °C for 20 s, 72 °C for 3 min, followed by a final 5 min extension at 72 °C.

Post-whole-transcriptome amplification proceeded as described above for SMART-Seq2 libraries, with the following exceptions: AMPure XP SPRI bead cleanup occurred first at a 0.6× volume ratio, followed by 0.8×. Library size was analyzed using an Agilent Tapestation hsD5000 Kit, confirming the expected peak at ~1000 bp, and absence of smaller peaks corresponding to primer. Libraries were quantified using the Qubit High-Sensitivity DNA Kit and prepared for Illumina sequencing using the Nextera XT DNA Sample Preparation Kit (Illumina) using 900 pg of cDNA library as input to tagmentation reactions. Amplified final libraries were purified twice with AMPure XP SPRI beads as before, with a volume ratio of 0.6× followed by 0.8×. Libraries from 3 Seq-Well arrays were pooled and sequenced together using a NextSeq 500/550 High Output v2 Kit (75 cycles) using a paired-end read structure with custom read 1 primer (Seq-Well CR1P; Supplementary Table 1): read 1: 20 bases, read 2: 50 bases, read 1 index: 8 bases.

### Seq-Well data processing for organoids and KP lung tumors

Reads were aligned and processed according to the Drop-Seq Computational Protocol v2.0 (https://github.com/broadinstitute/Drop-seq). Reads were first demultiplexed according to index read 1 using bcl2fastq (v2.17.1.14) with default settings. Read 1 was split into the first 12 bases corresponding to the cell barcode (CB), and the 13–20th bases, which encode the UMI. CBs, UMIs, and read 2 sequences with low base quality were discarded, as were any that contained non-random sequences (e.g., primer sequences, poly-A tails). Following CB and UMI tagging, read 2 was aligned to the mouse genome (version mm10) for all KP lung tumor experiments and to the human genome (version hg19) for all organoid experiments using STAR v2.5.2b with default parameters, including “—limitOutSJcollapsed 1000000—twopassMode Basic”. STAR alignments were merged to recover cell and molecular barcodes, and any sequences within hamming edit distance 1 were merged, as these likely originated from the same original sequence. Additional methods to correct for bead synthesis errors in the CB or UMI are detailed in the Drop-Seq Computational Protocol v2.0 (“DetectBeadSynthesisErrors” function). Digital gene expression matrices for each Seq-Well array were retained following quality filtering and UMI correction and further processed using the R language for Statistical Computing. Cells with <200 unique genes were removed from analysis.

### Seq-Well data analysis to identify cell types

Data from human organoid experiments and mouse KP lung tumors were processed similarly in R to visualize and identify cell types. Data were normalized and scaled using the Seurat R package (https://github.com/satijalab/seurat)^57^: transforming the data to log_e_(UMI + 1) and applying a scale factor of 10,000. We confirmed equivalent depth and cell quality across each of the KP lung tumor arrays and the absence of major batch effects introduced by sequencing work-up day or other technical factors and thus did not regress any batch-related covariates out of our data, including individual cell quality or mitochondrial gene expression. Organoid samples from our SPACECAT arrays along with samples from a previously existing, donor matched dataset were integrated as described below. To identify major axes of variation within our data, we first examined only highly variable genes across all cells, yielding approximately 1000–3000 variable genes with average expression >0.1 log-normalized UMI across all cells. An approximate PCA was applied to the cells to generate 100 principal components (PCs). Using the JackStraw function within Seurat, we identified significant PCs to be used for subsequent clustering and further dimensionality reduction^58^.

### Seq-Well data analysis of human organoid samples

After preprocessing and filtering, we recovered 266 cells from the Whole Organoid sample, 187 cells from the Calcein NVOC+ region, and 1541 and 1594 cells from our two reference datasets (genes/cell: 2197 ± 18, UMI/cell: 5595 ± 60, mean ± SEM) (Supplementary Data 1). To contextualize the organoid scRNA-seq data generated with the SPACECAT protocol, we merged the SPACECAT data with two donor-matched scRNA-seq reference datasets containing additional cells (*n* = 1541 and 1594 cells). To do so, we implemented normalization via Seurat’s negative binomial regression-based SCTransform with regression against cell cycle and cell quality-associated metrics (percentage of mitochondrial reads, number of unique molecules, and number of genes). We then integrated the two datasets using Seurat’s canonical correlation analysis-based method^59^. Clusters of transcriptionally similar cells were identified through unsupervised clustering with the Louvain algorithm^60^. Briefly, this method involves constructing a *k* nearest-neighbor graph over the Euclidean distance between cells in the PC reduced space, followed by a shared nearest neighbor-based clustering and modularity optimization^61^. We implemented this using the FindNeighbhors and FindClusters tool within the Seurat R package with default parameters, the first 10 PCs (by elbow plot of proportion of variance), and resolution set to 0.332 (based on maximizing silhouette coefficients of each cluster)^62^. Analysis of cell cycle stage was carried out using the Seurat function “CellCycleScoring” with default parameters (Fig. 2f, h). We used the Seurat function FindAllMarkers to identify differentially expressed genes upregulated within each cluster compared to all other cells in the dataset and tested differential expression using Wilcoxon test for single-cell gene expression^63^. The significantly differentially expressed genes (using a Bonferroni-corrected *p* value cutoff of 0.05) for each cluster were analyzed to attribute likely identities of cells in each cluster. Statistical significance of proportional differences by cell cycle stage or cell type was carried out using Chi-square test.

### Seq-Well data analysis of mouse KP lung tumors

After preprocessing and filtering, we recovered 2320 cells from Tumor 1, with 1634 of these cells coming from the Whole Tumor and 686 from the Healthy/Tumor Border. From Tumor 2, we recovered 2023 total cells, 1489 of which from the Whole Tumor and 534 from the Healthy/Tumor Border (Supplementary Data 3, genes/cell: 826 ± 11, UMI/cell: 2255 ± 37, mean ± SEM) (see Supplementary Note 1 for a discussion of power to detect rare cells). For 2D visualization and cell type clustering of the tumor data, we used a UMAP dimensionality reduction technique (https://github.com/lmcinnes/umap) with “min_dist” set to 0.5 and “n_neighbors” set to 30 (Fig. 4b, d, e). To identify clusters of transcriptionally similar cells, we employed unsupervised clustering as described above using the FindClusters tool within the Seurat R package with default parameters and k.param set to 10 and resolution set to 0.5. Here we intentionally underclustered our data to avoid erroneously splitting cells with shared cell-type functions, as the variable genes calculated for this dimensionally reduced space likely did not fully reflect more nuanced cell-type differences (e.g., variable behavior between lymphocyte subtypes). Each cluster was sub-clustered to identify more granular cell types, requiring each cell type to express >25 significantly upregulated genes by differential expression test (FindMarkers implemented in Seurat, setting “test.use” to “bimod” (a likelihood ratio test for single-cell expression^63^), Bonferroni-adjusted *p* value cutoff <0.001). After assessment of each subcluster, we identified 20 unique cell types across both tumors (Fig. 4b–e and Supplementary Data 3). Hierarchical clustering of cell types was conducted using complete linkage over the Spearman correlation between each cell type, computed over differentially expressed genes between each cell type and all other cell types (Fig. 4c). Differences in abundance of cell types by photoactivation region (Whole Tumor vs. Healthy/Tumor Border) was assessed by Fisher’s exact test with multiple hypothesis corrected using the Benjamini–Hochberg method (Fig. 4f).

Differential expression tests between cells from Whole Tumor samples vs. Healthy/Tumor Border samples within the Monocyte/Macrophage cluster were conducted using the likelihood ratio test for single-cell gene expression as described above (Fig. 4h, i). To quantify differences in subpopulation proportions within each tumor, Student’s *T* tests were conducted by comparing cell subpopulation proportions observed across the three separately processed Seq-Well arrays of Whole Tumor-derived cells against the single subpopulation proportion value from Healthy/Tumor Border-derived cells. To order Monocytes/Macrophages by their spatial distribution (Fig. 4j), we scored each cell by taking the sum of the 117 genes upregulated in Tumor 1 Monocytes/Macrophages found at the Healthy/Tumor Border and ranked single cells by this score. To identify gene programs associated with this ranking, we calculated the Pearson correlation between all genes and the Healthy/Tumor Border score. We used permutation testing to assess correlation values for significance, resulting in 321 genes negatively associated with the Healthy/Tumor Border phenotype and 360 genes positively associated with the Healthy/Tumor Border phenotype. Heatmap was generated using the ComplexHeatmap package^64^. Significantly enriched gene ontologies were assessed using the Database for Annotation, Visualization and Integrated Discovery (DAVID; Fig. 4k)^65^. All genes and statistics resulting from differential expression tests are reported in Supplementary Data 4.

### Statistics and reproducibility

Photoactivation experiments on cell lines and tissue sections were performed and replicated across multiple donors, species, tissue of origin, and experimental days. Photoactivation experiments on organoids were repeated across multiple days. Figure legends contain all details on replicate experiments and sample sizes for presented data, and all attempts at replication were successful.

### Reporting summary

Further information on research design is available in the Nature Research Reporting Summary linked to this article.

## Supplementary information

Supplementary Information

Supplementary Data 1

Supplementary Data 2

Supplementary Data 3

Supplementary Data 4

Reporting Summary

Description of Additional Supplementary Files

## Data Availability

The raw data and gene expression matrices for mouse tumor scRNA-seq data have been deposited in the Gene Expression Omnibus as GSE175882. For human intestinal organoids, interactive visualization tools, metadata, and digital gene expression matrices can be found through the Broad Institute’s Single-Cell Portal as study SCP1457. Digital gene expression matrices annotated with cell types, photoactivation regions, and other metadata can also be found in Supplementary Data 1 (human intestinal organoids, corresponding to Fig. 2) and Supplementary Data 3 (mouse KP lung tumors, corresponding to Fig. 4). Gene lists corresponding to differential expression tests for human intestinal organoids and mouse KP lung tumors can be found in Supplementary Data 2 and 4, respectively. FASTQ data for the human intestinal organoids is available upon request and with a data use agreement. Source data are provided with this paper.

## Source data

Source Data

## Code availability

Cell Profiler v 3.1.9 was used to segment microscopic images, and resulting data were analyzed using MATLAB R2020a. Sequencing data were demultiplexed using bl2fastq v2.17.1.14. Smart-Seq2 sequencing reads were aligned using STAR v2.5b and transcripts were quantified with RSEM v1.2.3. Seq-Well sequencing reads were aligned and processed according to the Drop-Seq Computational Protocol v2.0 (https://github.com/broadinstitute/Drop-seq). Single-cell RNA-seq analysis was performed in R version 4.0.2 using ggplot2 (v3.3.2130), Seurat (v4.0.1), and ComplexHeatmap (v2.7.3132). Gene ontologies were evaluated for enrichment using DAVID v6.8. Additional NMR processing was done with MestReNova (9.0.1) (Santiago de Compostela, Spain). MS data were processed using the MassHunter Workstation Software – Qualitative Analysis Version B.03.01 • build 3.1.346.6 • service pack 2 (Agilent Technologies). Code for analysis is available at https://github.com/ctzouanas/spacecat.

## References

[1] Tomura, M. et al. Monitoring cellular movement in vivo with photoconvertible fluorescence protein ‘Kaede’ transgenic mice. *Proc. Natl Acad. Sci. USA* **105**, 10871–10876 (2008). 10.1073/pnas.0802278105 PMC2504797 18663225

[2] Sato, T., Takahoko, M. & Okamoto, H. HuC:Kaede, a useful tool to label neural morphologies in networks in vivo. *Genesis* **44**, 136–142 (2006). 10.1002/gene.20196 16496337

[3] Stark, D. A. & Kulesa, P. M. An in vivo comparison of photoactivatable fluorescent proteins in an avian embryo model. *Dev. Dyn*. **236**, 1583–1594 (2007). 10.1002/dvdy.21174 17486622

[4] Medaglia, C. et al. Spatial reconstruction of immune niches by combining photoactivatable reporters and scRNA-seq. *Science* **358**, 1622–1626 (2017). 10.1126/science.aao4277 PMC7234837 29217582

[5] Chudakov, D. M., Matz, M. V., Lukyanov, S. & Lukyanov, K. A. Fluorescent proteins and their applications in imaging living cells and tissues. *Physiol. Rev.* **90**, 1103–1163 (2010). 10.1152/physrev.00038.2009 20664080

[6] Angelo, M. et al. Multiplexed ion beam imaging of human breast tumors. *Nat. Med*. **20**, 436–442 (2014). 10.1038/nm.3488 PMC4110905 24584119

[7] Lee, J. H. et al. Highly multiplexed subcellular RNA sequencing in situ. *Science* **343**, 1360–1363 (2014). 10.1126/science.1250212 PMC4140943 24578530

[8] Rodriques, S. G. et al. Slide-seq: a scalable technology for measuring genome-wide expression at high spatial resolution. *Science* **363**, 1463–1467 (2019). 10.1126/science.aaw1219 PMC6927209 30923225

[9] Satija, R., Farrell, J. A., Gennert, D., Schier, A. F. & Regev, A. Spatial reconstruction of single-cell gene expression data. *Nat. Biotechnol*. **33**, 495–502 (2015). 10.1038/nbt.3192 PMC4430369 25867923

[10] Halpern KB (2017). Single-cell spatial reconstruction reveals global division of labour in the mammalian liver. Nature.

[11] Weston, S. A. & Parish, C. R. New fluorescent dyes for lymphocyte migration studies. Analysis by flow cytometry and fluorescence microscopy. *J. Immunol. Methods* **133**, 87–97 (1990). 10.1016/0022-1759(90)90322-m 2212694

[12] Bourke S (2005). Development of a lung slice preparation for recording ion channel activity in alveolar epithelial type I cells. Respir. Res..

[13] Panel M, Ghaleh B, Morin D (2017). Ca2+ionophores are not suitable for inducing mPTP opening in murine isolated adult cardiac myocytes. Sci. Rep..

[14] Garnier D (2018). Expansion of human primary hepatocytes in vitro through their amplification as liver progenitors in a 3D organoid system. Sci. Rep..

[15] Wiederschain, G. Y. The Molecular Probes handbook. A guide to fluorescent probes and labeling technologies. *Biochemistry* **76**, 1276 (2011).

[16] Burdette, S. C., Walkup, G. K., Spingler, B., Tsien, R. Y. & Lippard, S. J. Fluorescent sensors for Zn2+ based on a fluorescein platform: synthesis, properties and intracellular distribution. *J. Am. Chem. Soc*. **123**, 7831–7841 (2001). 10.1021/ja010059l 11493056

[17] Jang YY (2012). An improved flow cytometry-based natural killer cytotoxicity assay involving calcein AM staining of effector cells. Ann. Clin. Lab. Sci..

[18] Waarsing JH (2004). Detecting and tracking local changes in the tibiae of individual rats: a novel method to analyse longitudinal in vivo micro-CT data. Bone.

[19] Grimm, J. B. et al. Bright photoactivatable fluorophores for single-molecule imaging. *Nat. Methods* **13**, 985–988 (2016). 10.1038/nmeth.4034 27776112

[20] Picelli S (2014). Full-length RNA-seq from single cells using Smart-seq2. Nat. Protoc..

[21] Trombetta, J. J. et al. Preparation of single-cell RNA-Seq libraries for next generation sequencing. *Curr. Protoc. Mol. Biol*. **107**, 4.22.1–4.22.17 (2014). 10.1002/0471142727.mb0422s107 PMC4338574 24984854

[22] Sato, T. et al. Long-term expansion of epithelial organoids from human colon, adenoma, adenocarcinoma, and Barrett’s epithelium. *Gastroenterology* **141**, 1762–1772 (2011). 10.1053/j.gastro.2011.07.050 21889923

[23] De Santa Barbara P, Van Den Brink GR, Roberts DJ (2003). Development and differentiation of the intestinal epithelium. Cell. Mol. Life Sci..

[24] Noah, T. K., Donahue, B. & Shroyer, N. F. Intestinal development and differentiation. *Exp. Cell Res.* **317**, 2702–2710 (2011). 10.1016/j.yexcr.2011.09.006 PMC3210330 21978911

[25] Gierahn TM (2017). Seq-Well: portable, low-cost RNA sequencing of single cells at high throughput. Nat. Methods.

[26] Aicher, T. P. et al. Seq-Well: a sample-efficient, portable picowell platform for massively parallel single-cell RNA sequencing. in *Methods in Molecular Biology* **1979**, 111–132 (2019). 10.1007/978-1-4939-9240-9_8 PMC7741456 31028635

[27] Hughes, T. K. et al. Second-Strand Synthesis-Based Massively Parallel scRNA-Seq Reveals Cellular States and Molecular Features of Human Inflammatory Skin Pathologies. *Immunity* **53**, 878–894.e7 (2020). 10.1016/j.immuni.2020.09.015 PMC7562821 33053333

[28] Scialdone, A. et al. Computational assignment of cell-cycle stage from single-cell transcriptome data. *Methods* **85**, 54–61 (2015). 10.1016/j.ymeth.2015.06.021 26142758

[29] Moor AE (2018). Spatial reconstruction of single enterocytes uncovers broad zonation along the intestinal villus axis. Cell.

[30] Haegebarth A, Clevers H (2009). Wnt signaling, Lgr5, and stem cells in the intestine and skin. Am. J. Pathol..

[31] Sato, T. et al. Single Lgr5 stem cells build crypt-villus structures in vitro without a mesenchymal niche. *Nature* **459**, 262–265 (2009). 10.1038/nature07935 19329995

[32] Sato T (2011). Long-term expansion of epithelial organoids from human colon, adenoma, adenocarcinoma, and Barrett’s epithelium. Gastroenterology.

[33] Liu R, Li H, Cai J, Wei Q, Han X (2019). Lgr5 + intestinal stem cell sorting and organoid culture. Anim. Model. Exp. Med..

[34] Ilicic T (2016). Classification of low quality cells from single-cell RNA-seq data. Genome Biol..

[35] Mebius, R. E. & Kraal, G. Structure and function of the spleen. *Nat. Rev. Immunol.* **5**, 606–616 (2005). 10.1038/nri1669 16056254

[36] Nolte, M. A., Hoen, E. N., Van Stijn, A., Kraal, G. & Mebius, R. E. Isolation of the intact white pulp. Quantitative and qualitative analysis of the cellular composition of the splenic compartments. *Eur. J. Immunol*. **30**, 626–634 (2000). 10.1002/1521-4141(200002)30:2<626::AID-IMMU626>3.0.CO;2-H 10671220

[37] DuPage, M., Dooley, A. L. & Jacks, T. Conditional mouse lung cancer models using adenoviral or lentiviral delivery of Cre recombinase. *Nat. Protoc*. **4**, 1064–1072 (2009). 10.1038/nprot.2009.95 PMC2757265 19561589

[38] McInnes, L., Healy, J., Saul, N. & Großberger, L. UMAP: uniform manifold approximation and projection. *J. Open Source Softw*. **3**, 861 (2018).

[39] LaFave LM (2020). Epigenomic state transitions characterize tumor progression in mouse lung adenocarcinoma. Cancer Cell.

[40] Marjanovic ND (2020). Emergence of a high-plasticity cell state during lung cancer evolution. Cancer Cell.

[41] Kimmerling, R. J. et al. Linking single-cell measurements of mass, growth rate, and gene expression. *Genome Biol*. **19**, 207 (2018). 10.1186/s13059-018-1576-0 PMC6260722 30482222

[42] Driehuis E, Kretzschmar K, Clevers H (2020). Establishment of patient-derived cancer organoids for drug-screening applications. Nat. Protoc..

[43] Wang, X. et al. Three-dimensional intact-tissue sequencing of single-cell transcriptional states. *Science* **361**, eaat5691 (2018). 10.1126/science.aat5691 PMC6339868 29930089

[44] Eng CHL (2019). Transcriptome-scale super-resolved imaging in tissues by RNA seqFISH. Nature.

[45] Xia C, Fan J, Emanuel G, Hao J, Zhuang X (2019). Spatial transcriptome profiling by MERFISH reveals subcellular RNA compartmentalization and cell cycle-dependent gene expression. Proc. Natl Acad. Sci. USA.

[46] Stickels, R. R. et al. Highly sensitive spatial transcriptomics at near-cellular resolution with Slide-seqV2. *Nat. Biotechnol*. **39**, 313–319 (2021). 10.1038/s41587-020-0739-1 PMC8606189 33288904

[47] Vickovic S (2019). High-definition spatial transcriptomics for in situ tissue profiling. Nat. Methods.

[48] Waylen LN, Nim HT, Martelotto LG, Ramialison M (2020). From whole-mount to single-cell spatial assessment of gene expression in 3D. Commun. Biol..

[49] Asp M, Bergenstråhle J, Lundeberg J (2020). Spatially resolved transcriptomes—next generation tools for tissue exploration. Bioessays.

[50] Hu KH (2020). ZipSeq: barcoding for real-time mapping of single cell transcriptomes. Nature.

[51] van der Leun, A. M. et al. Single cell analysis of regions of interest (SCARI) using a novel photoswitchable tag. Preprint at *bioRxiv*10.1101/2020.10.02.291096 (2020).

[52] Lippard, S. J. & Woodroofe, C. C. Sensors, and methods of making and using the same. US20040224420A1. https://patents.google.com/patent/US20040224420 (2004).

[53] Li, W. & Li, D. Zinc sensors for cellular imaging. US8530183B2. https://patents.google.com/patent/US8530183B2/en (2013).

[54] Fujii M (2018). Human intestinal organoids maintain self-renewal capacity and cellular diversity in niche-inspired culture condition. Cell Stem Cell.

[55] McQuin C (2018). CellProfiler 3.0: next-generation image processing for biology. PLoS Biol..

[56] Dobin, A. et al. STAR: ultrafast universal RNA-seq aligner. *Bioinformatics* **29**, 15–21 (2013). 10.1093/bioinformatics/bts635 PMC3530905 23104886

[57] Butler, A., Hoffman, P., Smibert, P., Papalexi, E. & Satija, R. Integrating single-cell transcriptomic data across different conditions, technologies, and species. *Nat. Biotechnol*. **36**, 411–420 (2018). 10.1038/nbt.4096 PMC6700744 29608179

[58] Shekhar, K. et al. Comprehensive classification of retinal bipolar neurons by single-cell transcriptomics. *Cell* **166**, 1308.e30–1323.e30 (2016). 10.1016/j.cell.2016.07.054 PMC5003425 27565351

[59] Stuart T (2019). Comprehensive integration of single-cell data. Cell.

[60] Blondel VD, Guillaume JL, Lambiotte R, Lefebvre E (2008). Fast unfolding of communities in large networks. J. Stat. Mech. Theory Exp..

[61] Waltman, L. & Van Eck, N. J. A smart local moving algorithm for large-scale modularity-based community detection. *Eur. Phys. J. B* **86**, 471 (2013).

[62] Ziegler CGK (2020). SARS-CoV-2 receptor ACE2 is an interferon-stimulated gene in human airway epithelial cells and is detected in specific cell subsets across tissues. Cell.

[63] McDavid, A. et al. Data exploration, quality control and testing in single-cell qPCR-based gene expression experiments. *Bioinformatics* **29**, 461–467 (2013). 10.1093/bioinformatics/bts714 PMC3570210 23267174

[64] Gu Z, Eils R, Schlesner M (2016). Complex heatmaps reveal patterns and correlations in multidimensional genomic data. Bioinformatics.

[65] Huang, D. W., Sherman, B. T. & Lempicki, R. A. Systematic and integrative analysis of large gene lists using DAVID bioinformatics resources. *Nat. Protoc*. **4**, 44–57 (2009). 10.1038/nprot.2008.211 19131956

[66] Shalek AK (2014). Single-cell RNA-seq reveals dynamic paracrine control of cellular variation. Nature.

[67] Yin X (2016). Engineering stem cell organoids. Cell Stem Cell.

[68] Tirosh, I. et al. Dissecting the multicellular ecosystem of metastatic melanoma by single-cell RNA-seq. *Science* **352**, 189–196 (2016). 10.1126/science.aad0501 PMC4944528 27124452

