# Publisher Correction: Live cell tagging tracking and isolation for spatial transcriptomics using photoactivatable cell dyes

**DOI:** 10.1038/s41467-021-25672-7

**Published:** 2021-09-03

**Authors:** Alex S. Genshaft, Carly G. K. Ziegler, Constantine N. Tzouanas, Benjamin E. Mead, Alex M. Jaeger, Andrew W. Navia, Ryan P. King, Miyeko D. Mana, Siyi Huang, Vanessa Mitsialis, Scott B. Snapper, Ömer H. Yilmaz, Tyler Jacks, Jeffrey F. Van Humbeck, Alex K. Shalek

**Affiliations:** 1 Institute for Medical Engineering & Science, MIT, Cambridge, MA USA; 2 Department of Chemistry, MIT, Cambridge, MA USA; 3 Koch Institute for Integrative Cancer Research, MIT, Cambridge, MA USA; 4 The Ragon Institute of MGH, MIT and Harvard, Cambridge, MA USA; 5 Broad Institute of MIT and Harvard, Cambridge, MA USA; 6 Harvard-MIT Program in Health Sciences and Technology, Harvard Medical School, Cambridge, MA USA; 7 Department of Immunology & HMS Center for Immune Imaging, Harvard Medical School, Boston, MA USA; 8 Division of Gastroenterology, Hepatology, and Nutrition, Boston Children’s Hospital, Boston, MA USA; 9 Division of Gastroenterology, Brigham and Women’s Hospital, Boston, MA USA; 10 Department of Biology, Massachusetts Institute of Technology, Cambridge, MA USA; 11 Department of Pathology, Massachusetts General Hospital and Harvard Medical School, Boston, MA USA; 12 Howard Hughes Medical Institute, Chevy Chase, MD USA; 13 Department of Chemistry, University of Calgary, Calgary, AB Canada

**Keywords:** RNA sequencing, Assay systems, Transcriptomics, Multicellular systems

Correction to: *Nature Communications* 10.1038/s41467-021-25279-y, published online 17 August 2021.

The original version of this Article contained an error in Fig. 3C furthest right panel, in which two white dots were inadvertently introduced. This has been corrected in both the PDF and HTML versions of the Article.

