# Supplementary material for: Live cell tagging tracking and isolation for spatial transcriptomics using photoactivatable cell dyes
Source: Nat Commun. 2021 Aug 17;12:4995. doi: 10.1038/s41467-021-25279-y (PMC8371137; doi:10.1038/s41467-021-25279-y)
Supplement: Supplementary file 1 — Supplementary Information [file 41467_2021_25279_MOESM1_ESM.pdf]

Supplementary Information

Live Cell Tagging Tracking and Isolation for Spatial Transcriptomics Using Photoactivatable Cell Dyes

Alex S Genshaft[1,2,3,4,5,6,*], Carly G. K. Ziegler[1,2,3,4,5,6,*], Constantine N. Tzouanas[1,2,3,4,5,6,*], Benjamin E. Mead[1,2,3,4,5,6], Alex M. Jaeger[3], Andrew W. Navia[1,2,3,4,5,6], Ryan P. King[2], Miyeko D. Mana[3], Siyi Huang[4,7], Vanessa Mitsialis[8,9], Scott B. Snapper[8,9], Ömer H. Yilmaz[3,5,10,11], Tyler Jacks[3,12], Jeffrey F. Van Humbeck[13], Alex K. Shalek[1,2,3,4,5,6,#]

AFFILIATIONS:

[1] Institute for Medical Engineering & Science, MIT, Cambridge, MA 02139, USA

[2] Department of Chemistry, MIT, Cambridge, MA 02139, USA

[3] Koch Institute for Integrative Cancer Research, MIT, Cambridge, MA 02139, USA

[4] The Ragon Institute of MGH, MIT and Harvard, Cambridge, MA 02139, USA

[5] Broad Institute of MIT and Harvard, Cambridge, MA 02142, USA

[6] Harvard-MIT Program in Health Sciences and Technology, Harvard Medical School, Cambridge, MA 02139, USA

[7] Department of Immunology & HMS Center for Immune Imaging, Harvard Medical School, Boston, MA 02115, USA

[8] Division of Gastroenterology, Hepatology, and Nutrition, Boston Children's Hospital, Boston, MA 02115, USA

[9] Division of Gastroenterology, Brigham and Women's Hospital, Boston, MA 02115, USA

[10] Department of Biology, Massachusetts Institute of Technology, Cambridge, MA 02139, USA

[11] Department of Pathology, Massachusetts General Hospital and Harvard Medical School, Boston, MA 02114, USA

[12] Howard Hughes Medical Institute, Chevy Chase, MD 20815, USA

[13] Department of Chemistry, University of Calgary, Calgary, AB, T2N1N4, Canada

[*] These authors contributed equally to this work

[#] To whom correspondence should be addressed: shalek@mit.edu (A.K.S.)

Supplementary Figures:

Supplementary Figure 1. Overview of SPACECAT applications and chemistry.
Supplementary Figure 2. NMR and HRMS of molecule (1)
Supplementary Figure 3. NMR and HRMS of molecule (2)
Supplementary Figure 4. NMR and HRMS of molecule (3)
Supplementary Figure 5. NMR and HRMS of molecule (4)
Supplementary Figure 6. Multiplexing photo-activatable probes
Supplementary Figure 7. Testing SPACECAT precision by specific phototagging of co-cultured HEK293T and NIH/3T3 cells.
Supplementary Figure 8. Characterization of SPACECAT photoactivation in Z-dimension and flow sorting.
Supplementary Figure 9. Application of SPACECAT for spatial tagging in tissue sections across health and disease.

Supplementary Tables:

Supplementary Table 1. Primer names and sequences used for Smart-Seq2 and Seq-Well scRNA-seq.

Supplementary Notes:

Supplementary Note 1. Power analysis to detect rare cell populations

Supplementary Data:

Supplementary Data 1. Digital gene expression matrix and metadata for cells derived from human intestinal organoids.
Supplementary Data 2. Differentially expressed genes between photoactivated and non-photoactivated control “Stem.1” cells.
Supplementary Data 3. Digital gene expression matrix and metadata for cells derived from mouse KP lung tumors.
Supplementary Data 4. Differentially expressed genes by cell type and tumor location in KP autochthonous lung tumors.

Supplementary Figure 1

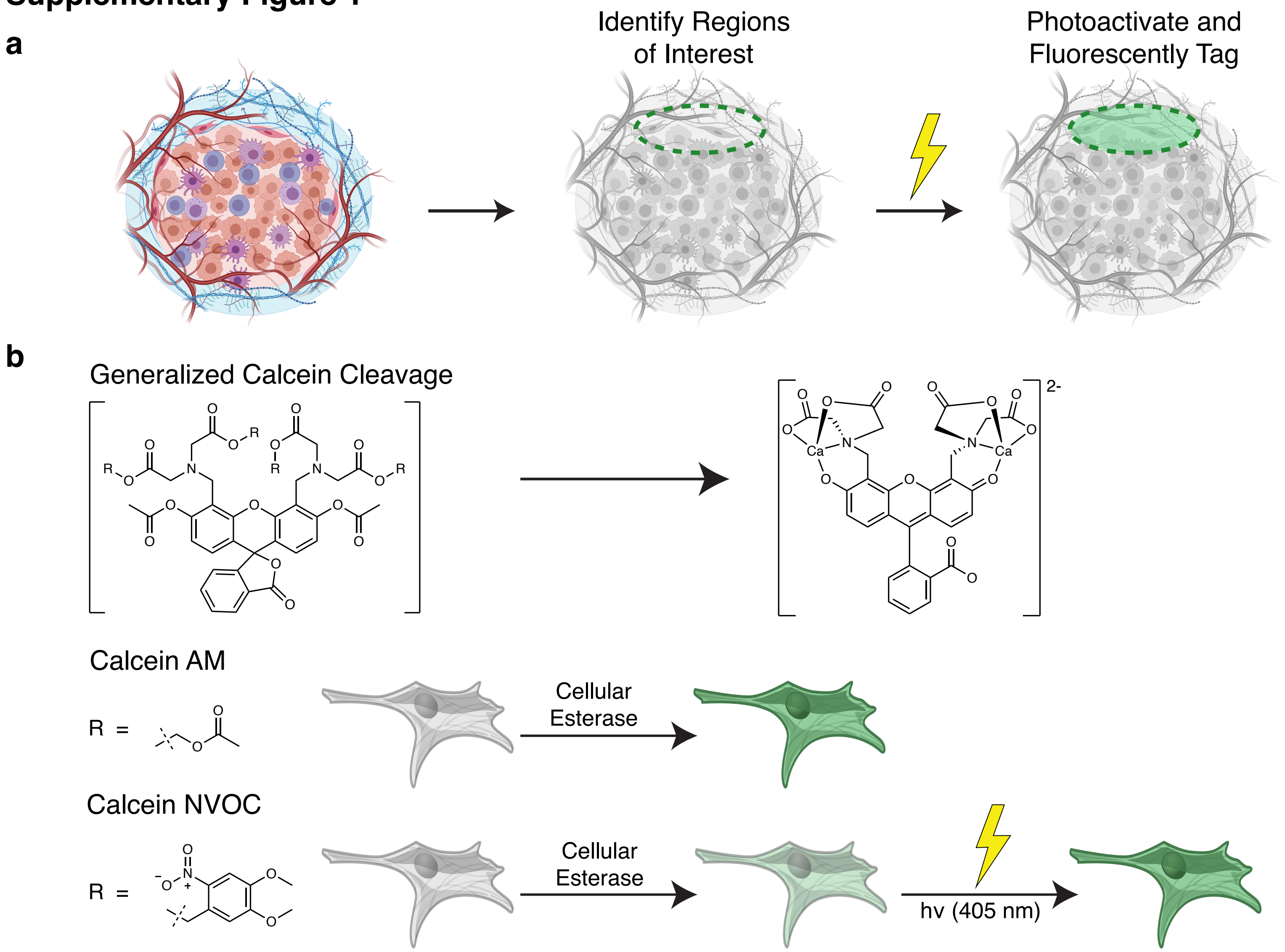

Supplementary Figure 1. Overview of SPACECAT applications and chemistry.
a. Schematic: application of SPACECAT to record spatial information as fluorescence signals in an arbitrary region of interest in complex primary tissue. Adapted from "Tumor extracellular matrix reduces therapeutic efficiency in solid tumors" by BioRender.com (2021). **b.** Chemical structure of calcein NVOC and standard calcein AM and their respective intracellular fluorescence transformations.

Supplementary Figure 2

a

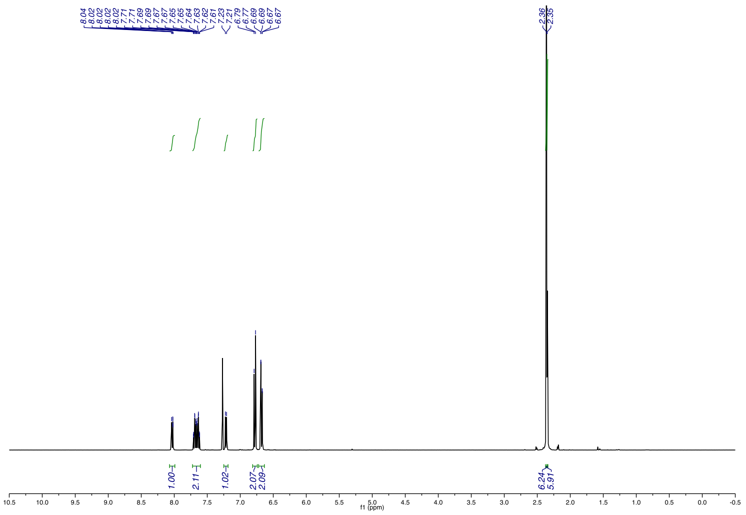

^{1}H NMR of 4’,5’-Dimethyl-3’6’-diacetoxyspiro[isobenzofuran-1(3*H*),9’-[9*H*]xanthen-3-one (**1**)

b

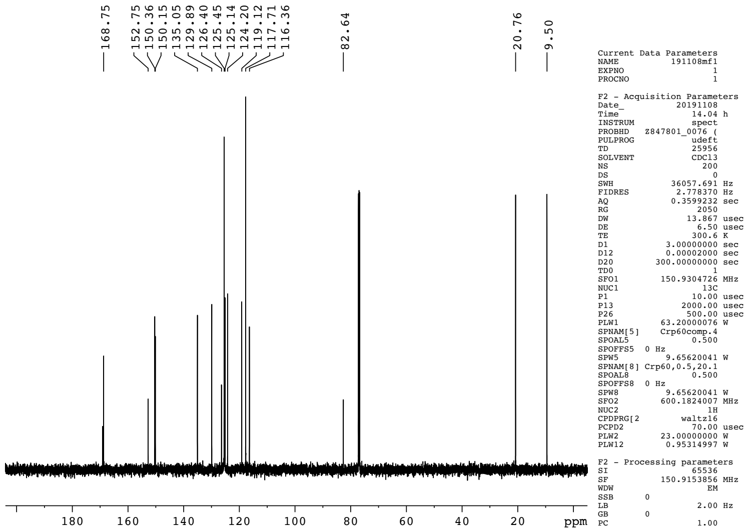

^{13}C NMR of 4’,5’-Dimethyl-3’6’-diacetoxyspiro[isobenzofuran-1(3*H*),9’-[9*H*]xanthen-3-one (**1**)

c

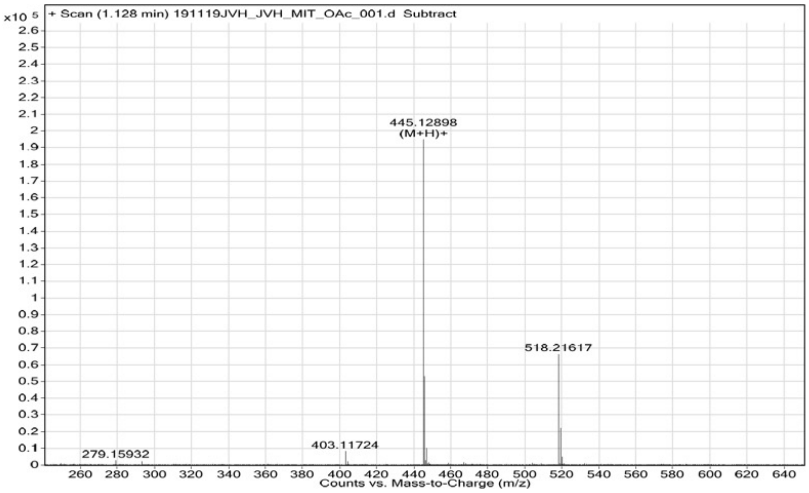

HRMS of 4’,5’-Dimethyl-3’6’-diacetoxyspiro[isobenzofuran-1(3*H*),9’-[9*H*]xanthen-3-one (**1**)

Supplementary Figure 2. NMR and HRMS of molecule (1)
^{1}H NMR (**a**), ^{13}C NMR (**b**), and HRMS (**c**) of 4',5'-Dimethyl-3'6'-diacetoxyspiro[isobenzofuran-1(3*H*),9'-[9*H*]xanthen-3-one (**1**).

Supplementary Figure 3

a

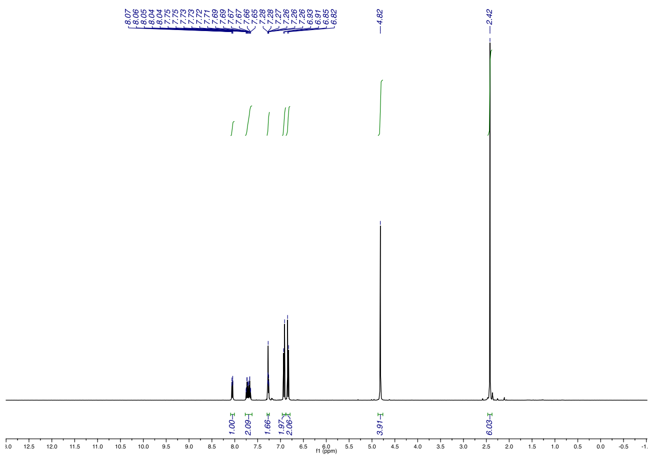

^{1}H NMR of 4’,5’-Bis-(bromomethyl)-3’6’-diacetoxyspiro[isobenzofuran-1(3*H*),9’-[9*H*]xanthen-3-one (**2**)

b

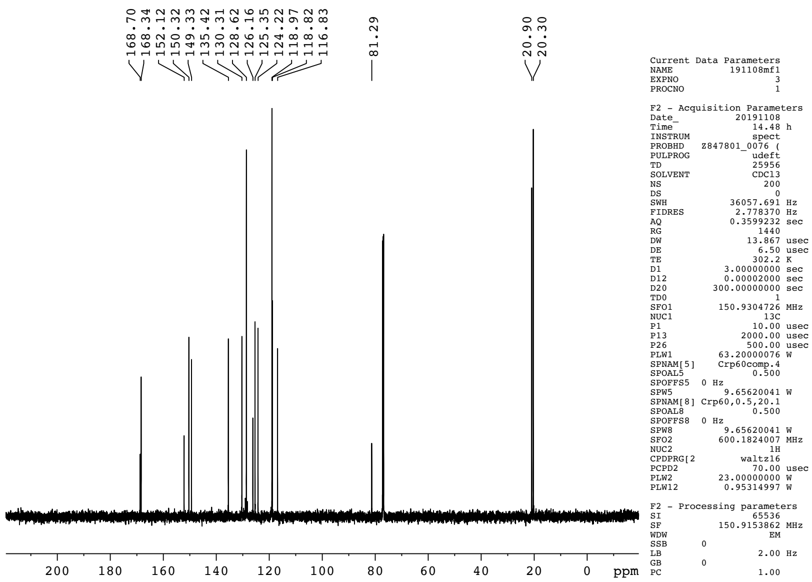

^{13}C NMR of 4’,5’-Bis-(bromomethyl)-3’6’-diacetoxyspiro[isobenzofuran-1(3*H*),9’-[9*H*]xanthen-3-one (**2**).

c

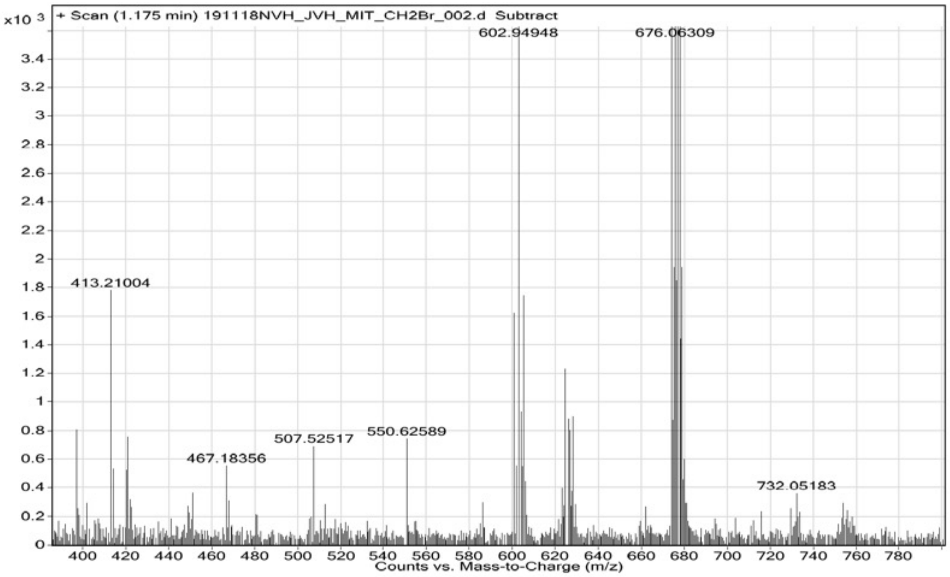

HRMS of 4’,5’-Bis-(bromomethyl)-3’6’-diacetoxyspiro[isobenzofuran-1(3*H*),9’-[9*H*]xanthen-3-one (**2**).

Supplementary Figure 3. NMR and HRMS of molecule (2)

^{1}H NMR (**a**), ^{13}C NMR (**b**), and HRMS (**c**) of 4',5'-Bis-(bromomethyl)-3'6'-diacetoxyspiro[isobenzofuran-1(3*H*),9'-[9*H*]xanthen-3-one (**2**).

Supplementary Figure 4

a

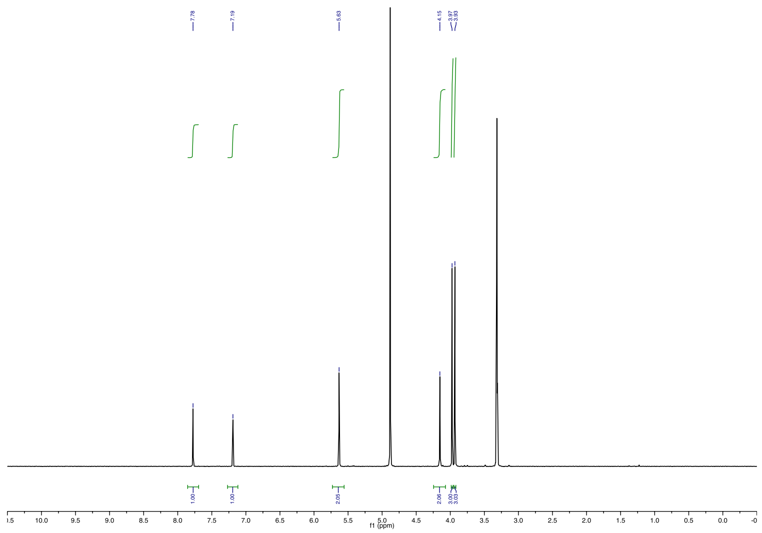

^{1}H NMR of Iminodiacetic acid bis(2-nitro-4,5-dimethoxy)benzyl ester trifluoroacetate salt (**3**)

b

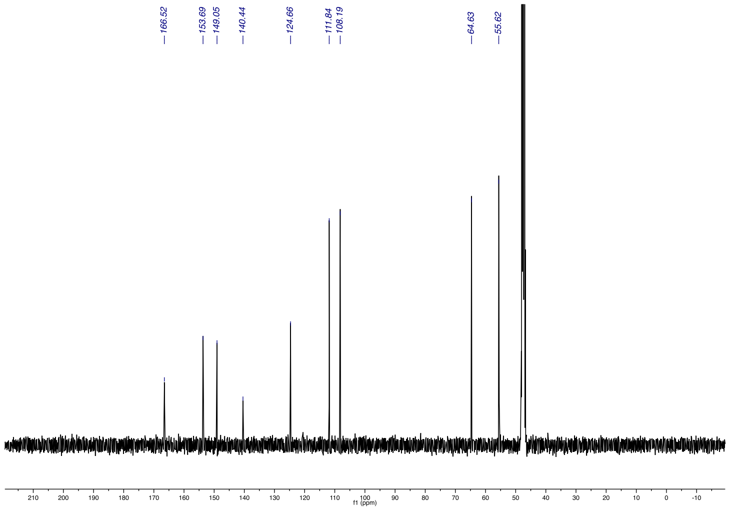

^{13}C NMR of Iminodiacetic acid bis(2-nitro-4,5-dimethoxy)benzyl ester trifluoroacetate salt (**3**)

c

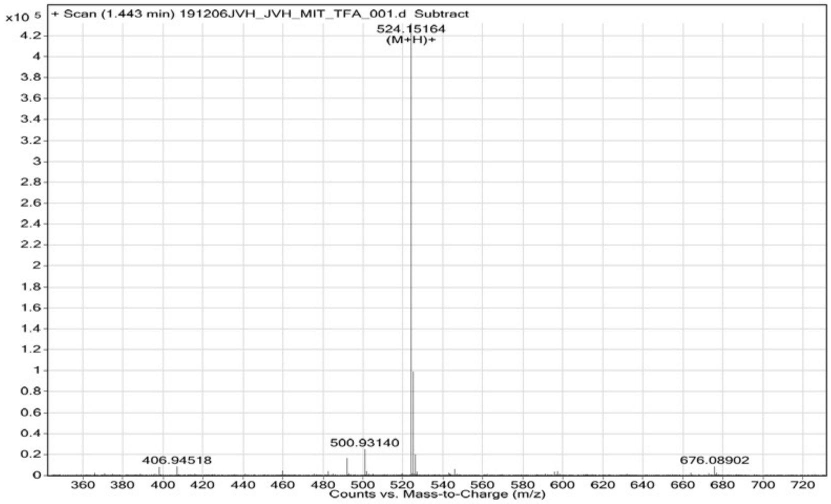

HRMS of Iminodiacetic acid bis(2-nitro-4,5-dimethoxy)benzyl ester trifluoroacetate salt (**3**)

Supplementary Figure 4. NMR and HRMS of molecule (3)

^{1}H NMR (**a**), ^{13}C NMR (**b**), and HRMS (**c**)of Iminodiacetic acid bis(2-nitro-4,5-dimethoxy)benzyl ester trifluoroacetate salt (**3**).

Supplementary Figure 5

a

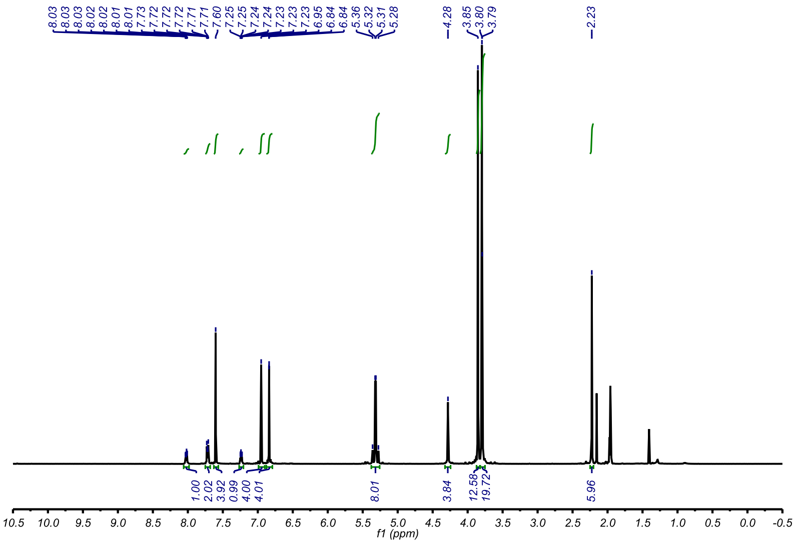

^{1}H NMR of calcein NVOC (**4**)

b

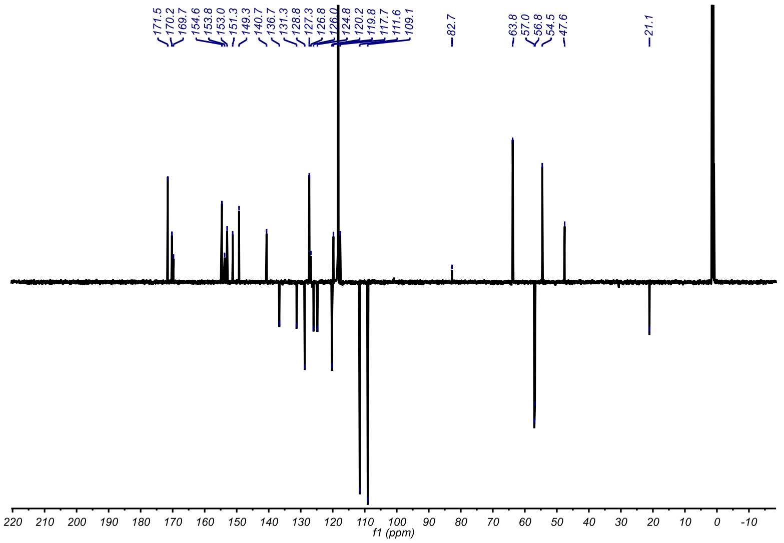

^{13}C NMR of calcein NVOC (**4**)

c

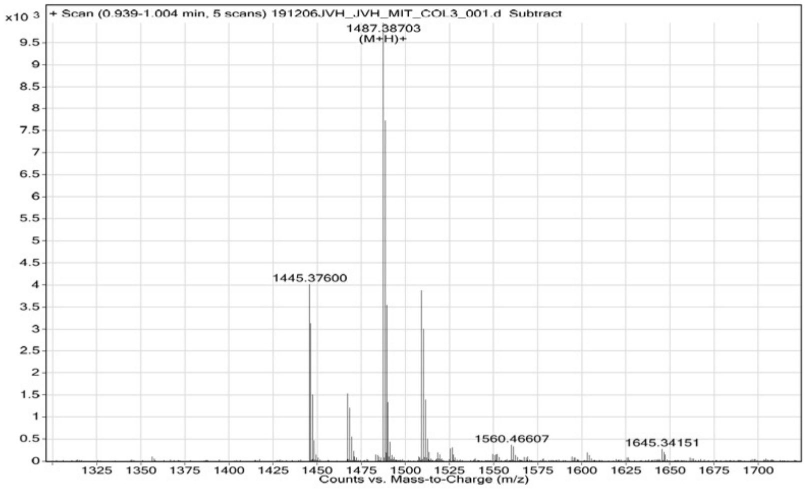

HRMS of calcein NVOC (**4**)

Supplementary Figure 5. NMR and HRMS of molecule (4)
^{1}H NMR (**a**), ^{13}C NMR (**b**), and HRMS (**c**) of Calcein NVOC (**4**).

Supplementary Figure 6

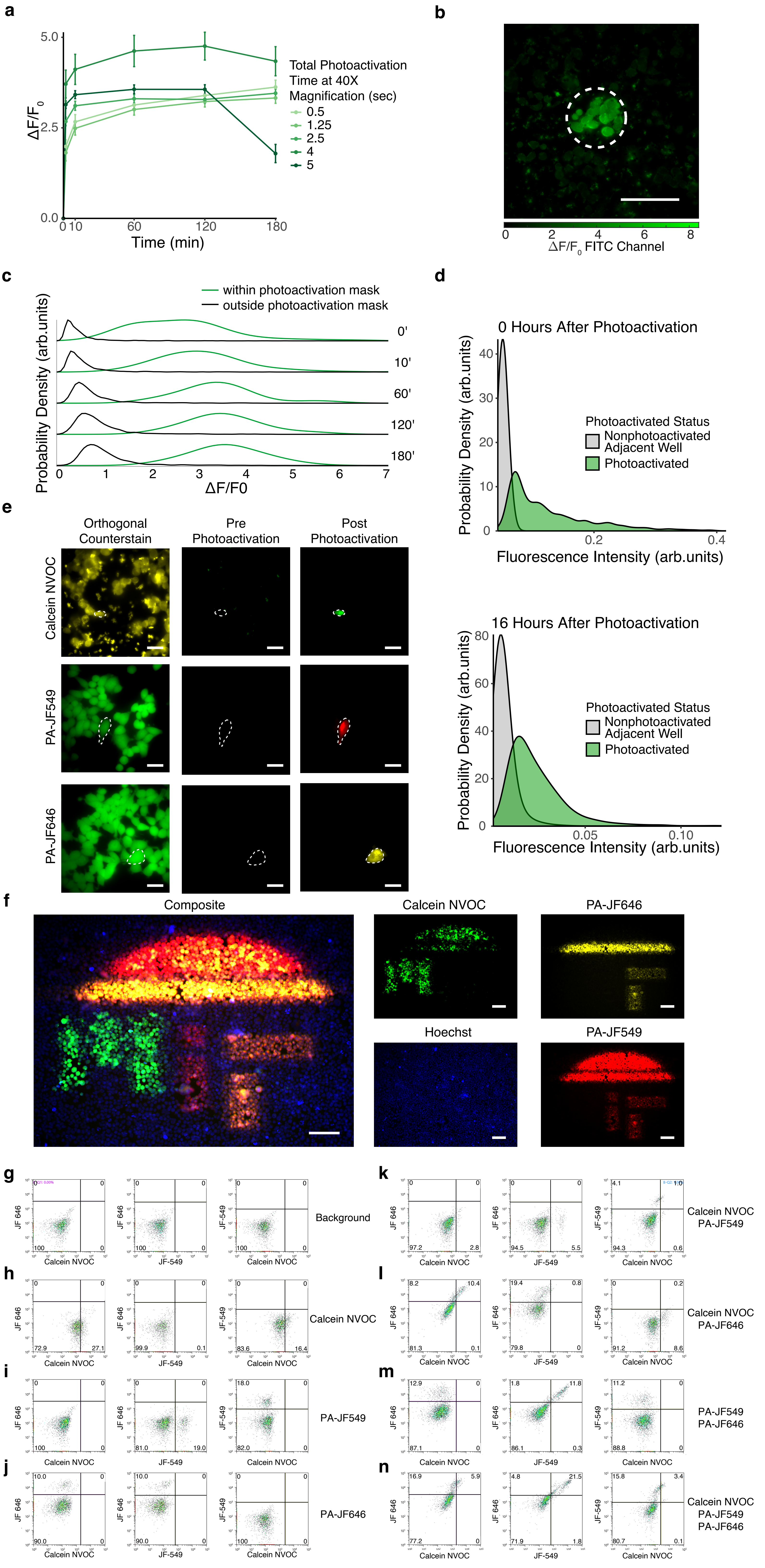

Supplementary Figure 6. Multiplexing photo-activatable probes

a Effects of photoactivation exposure time on cellular viability and calcein NVOC fluorescence signal magnitude (n = 13 cells across 2 fields of view for 0.5 sec. exposure; n = 14 cells across 2 fields of view for 1.25 sec. exposure; n = 21 cells across 2 fields of view for 2.5 sec. exposure; n = 13 cells across 2 fields of view for 4 sec. exposure; n = 20 cells across 2 fields of view for 5 sec. exposure). **b** $\Delta F/F_0$ for cells shown in Fig 1c (which is one of the independent fields of view with 4 sec. exposure shown in Supplementary Fig. 6a). Scale bar = 100 μm. **c** Distributions of fluorescence changes across a 3 h timecourse for cells outside of photoactivated regions (grey, n = 1,347 cells across 8 fields of view) and inside photoactivated regions (green, n = 68 cells across 8 fields of view). **d** Calcein NVOC fluorescence levels in photoactivated cell (green) vs. cells in non-photoactivated adjacent wells (grey) immediately after photoactivation (top; n = 1,066 photoactivated cells across 11 fields of view; 2,427 non-photoactivated cells across 5 fields of view) or 16 h after photoactivation (bottom; n = 3,691 photoactivated cells across 10 fields of view; 15,571 cells across 5 fields of view). **e** Images showing single-cell photoactivation using calcein NVOC (top row), PA-JF549 (middle row) and PA-JF646 (bottom row). Scale bar = 50 μm. n = 3 independent fields of view for each photoactivatable fluorescent dye. **f** Representative images of color multiplexing by photoactivating arbitrary regions in HEK293T cells using sequential addition of 3 photoactivatable probes (calcein NVOC, PA-JF549, PA-JF646) and different photoactivation thresholds (10 s for calcein NVOC, 0.5 s for PA-JF549 and PA-JF646). Scale bar = 100 μm. Multiplexed encoding scheme repeated a total of n = 2 times. **g-n** HEK293T cells were incubated with different combinations of photoactivatable probes (rows) and ~2-20% of all cells in each sample were exposed to near-UV light, inducing the observed fluorescence increases. Cells were dissociated, exposed to a master mix of all 3 dyes (to account for background fluorescence from individual dyes), and analyzed by flow cytometry. Gates determined by cells in control sample well stained with all dyes without UV-excitation. Left: calcein NVOC vs. PA-JF646, middle: PA-JF549 vs. PA-JF646, right: calcein NVOC vs. PA-JF549. Numbers reflect percent of cells within each quadrant. **g** photoactivated without photo-convertible dyes (10 s for calcein NVOC, 0.5 s for PA-JF549 and PA-JF646), **h** photoactivated while exposed to calcein NVOC only, **i** PA-JF549 only, **j** PA-JF646 only, **k** calcein NVOC and PA-JF549, **l** calcein NVOC and PA-JF646, **m** PA-JF549 and PA-JF646, **n** calcein NVOC, PA-JF549, PA-JF646.

Supplementary Figure 7

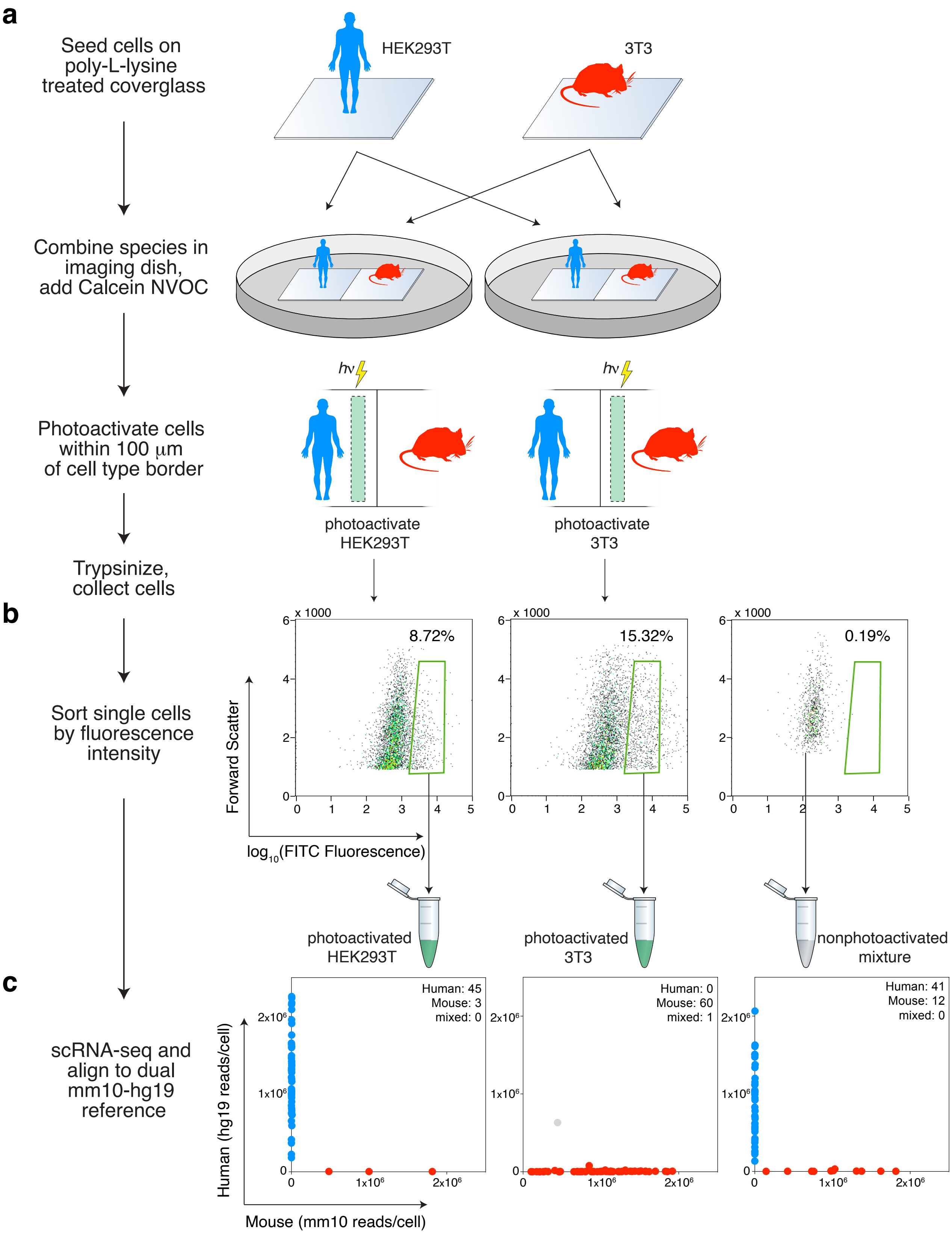

Supplementary Figure 7. Testing SPACECAT precision by specific phototagging of co-cultured HEK293T and NIH/3T3 cells. a. Schematic of modified species-mixing experiment to test precision of spatially-restricted photoactivation. Human (HEK293T) and mouse (NIH/3T3) cell lines were seeded on separate coverglass slides and placed abutting in an imaging dish with calcein NVOC. **b.** FACS analysis and sort gates. Three such dishes (and corresponding FACS samples) were created: (left) HEK293T cells photoactivated, (center) NIH/3T3 cells photoactivated, and (right) non-photoactivated. **c.** SMART-Seq2 library alignments to dual mm10-hg19 reference genome. Points represent single cells plotted by number of reads aligned to mm10 (x-axis) or hg19 (y-axis). Red: alignments support mouse cell, blue: alignments support human cell, grey: indeterminate species by transcriptome alignment.

Supplementary Figure 8

a

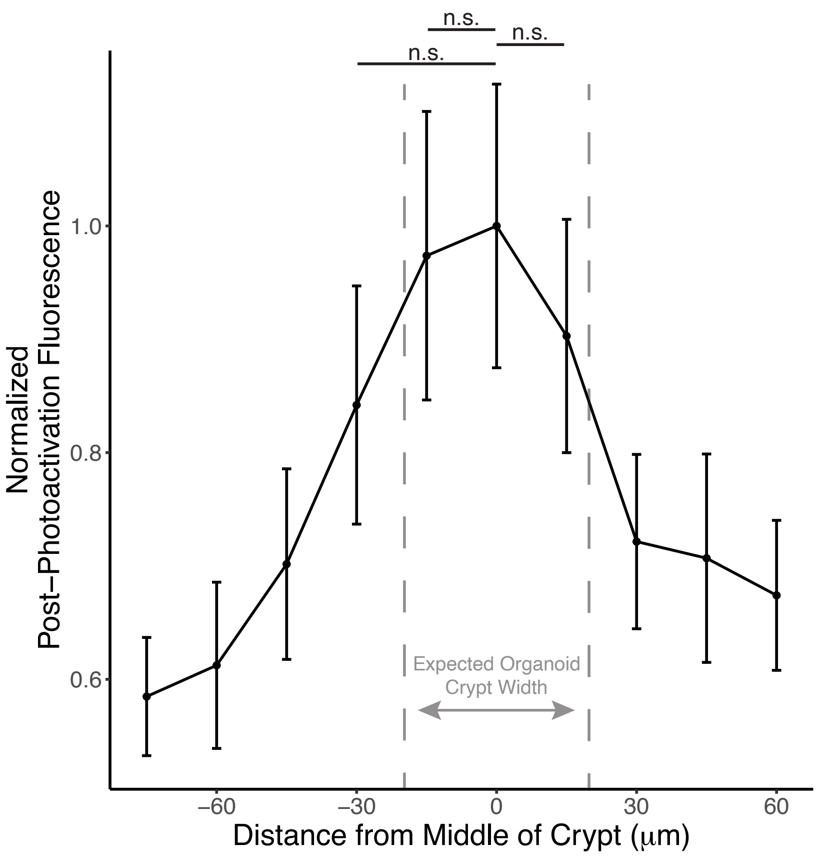

b

Distance from Middle of Crypt	Cohen's d vs. Middle of Crypt	P-value vs. Middle of Crypt
-75	1.09	0.007
-60	1.00	0.01
-45	0.75	0.06
-30	0.37	0.34
-15	0.06	0.88
0	0	1
15	0.23	0.55
30	0.72	0.07
45	0.71	0.07
60	0.74	0.03

c

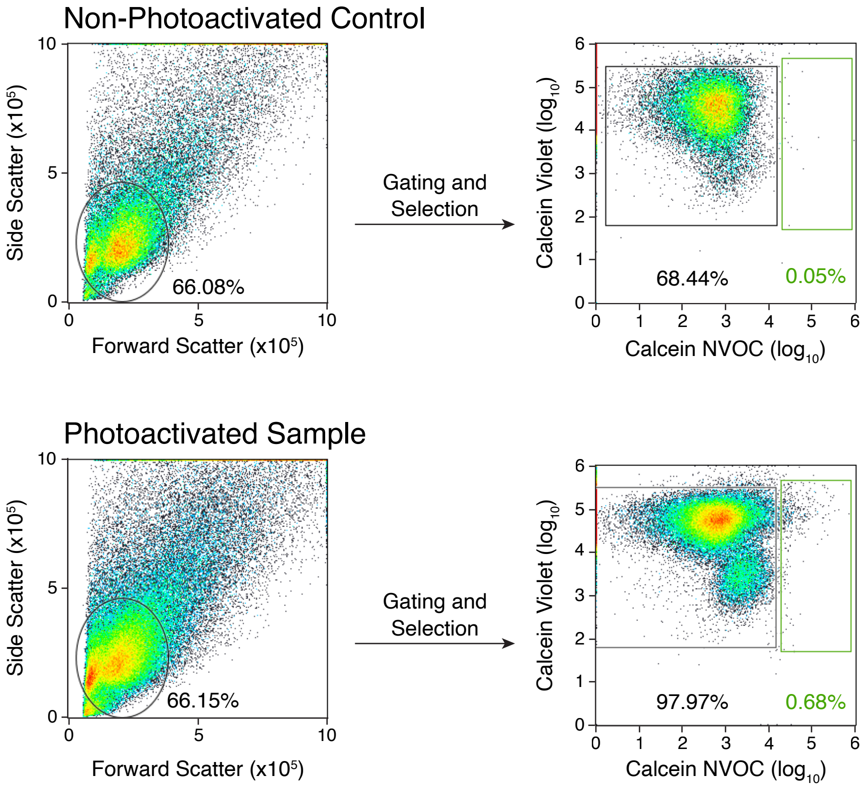

Supplementary Figure 8. Characterization of SPACECAT photoactivation in Z-dimension and flow sorting. a Post-photoactivation fluorescence levels of z-stack images of human intestinal organoid crypts, normalized against the fluorescence level at the middle of each crypt (plotted are mean ± SEM of 14 crypts across 10 organoids). Fluorescence levels at z = -30 μm, -15 μm, and +15 μm are not statistically significantly different from fluorescence levels at z = 0 μm, while fluorescence levels from other focal planes are statistically significantly different (two-sided Student's T test, $\alpha = 0.1$). **b** Effect sizes and p-values of differences in fluorescence levels between each frame of the organoid z-stack and the fluorescence level of the middle of each crypt, calculated as Cohen's d and two-sided Student's T test, respectively. **c** Flow gating and sorting scheme for non-photoactivated and photoactivated human intestinal organoid samples used to generate single-cell RNA-seq data presented in fig. 2.

Supplementary Figure 9

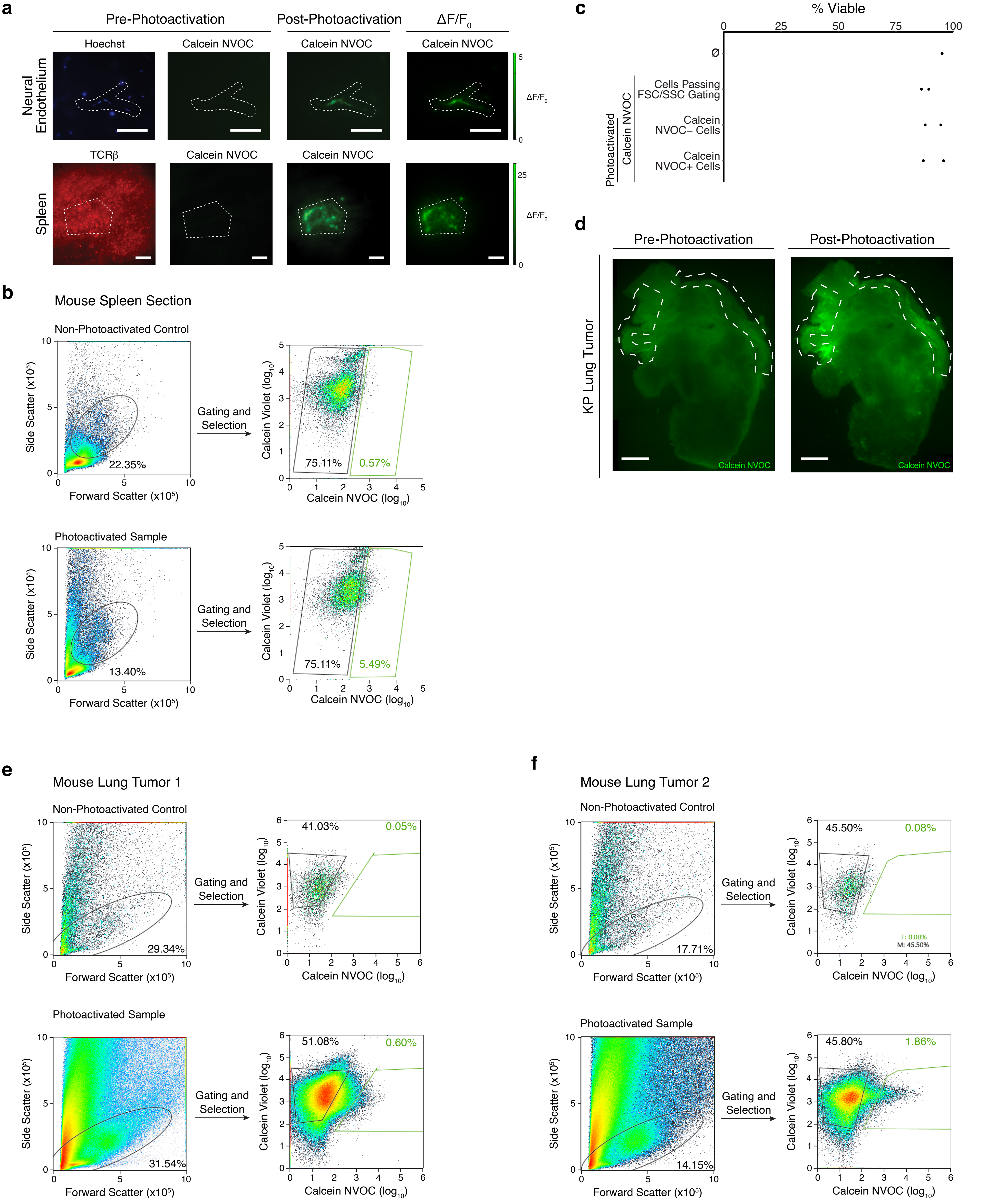

Supplementary Figure 9. Application of SPACECAT for spatial tagging in tissue sections across health and disease. a Representative images of mouse brain and spleen samples stained with calcein NVOC and photoactivated in arbitrary regions (within white dashed boundaries). Scale bars = 100 μm. n = 2 independent fields of view from brain samples. n = 3 independent fields of view for spleen samples. **b** Flow gating scheme for non-photoactivated and photoactivated mouse spleen sections used to generate single-cell RNA-seq data presented in fig. 3e-h. **c** Cell percent viability by calcein violet staining after photoactivation. N=1 lung tumor sample for the null condition, N = 2 lung tumor samples for all other conditions. **d.** Photoactivation of Healthy/Tumor Border of KP lung Tumor 2. Scale bars = 500 μm. n = 2 independent tumor sections. **e** Flow gating scheme for non-photoactivated and photoactivated sections from lung tumor 1 used to generate single-cell RNA-seq data presented in fig. 4. **f** Flow gating scheme for non-photoactivated and photoactivated sections from lung tumor 2 used to generate single-cell RNA-seq data presented in fig. 4.

Supplementary Table 1: Primer names and sequences used for Smart-Seq2 and Seq-Well scRNA-seq

**Primer Name**	**Sequence**
Seq-Well Template Switch Oligo (SW-TSO)	AAGCAGTGGTATCAACGCAGAGTGAATrGrGrG
dN-SMRT Oligo	AAGCAGTGGTATCAACGCAGAGTGANNNGGNNNB
ISPCR primer	AAGCAGTGGTATCAACGCAGAGT
Custom Read 1 Primer (CR1P)	GCCTGTCCGCGGAAGCAGTGGTATCAACGCAGAGTAC
oligo-dT Smart-Seq2 RT primer	/5Biosg/AAGCAGTGGTATCAACGCAGAGTACTTTTTTTTTTTTTTTTTTTTTTTTTTTTTTVN
Smart-Seq2 Template Switch Oligo	AAGCAGTGGTATCAACGCAGAGTACATrGrGrG

Supplementary Note 1

In our application of SPACECAT to mouse KP lung tumors (Fig. 4), we recovered 1,634 cells from the Whole Tumor 1, 686 cells from the Healthy/Tumor 1 Border, 1,489 cells from Whole Tumor 2, and 534 cells from the Healthy/Tumor 2 Border.

With cell counts from various tumor samples on the order of 500-1,500 cells, this brief power analysis evaluates our ability to detect rare cell populations and differences in proportions. Given an $\alpha = 0.05$ and 10 “positive” cells as a threshold value for being able to identify a cluster of “positive” rare cells, our sample sizes allow us power to detect subpopulations as rare as 1.7% (i.e., α = P(detecting ≤ 10 cells from a rare cell subpopulation) = BinomialCDF(10 successes, p = 0.017, 1000 trials) = 0.0478).
