# Supplementary material for: Live cell tagging tracking and isolation for spatial transcriptomics using photoactivatable cell dyes
Source: Nat Commun. 2021 Aug 17;12:4995. doi: 10.1038/s41467-021-25279-y (PMC8371137; doi:10.1038/s41467-021-25279-y)
Supplement: Supplementary file 6 — Reporting Summary [file 41467_2021_25279_MOESM6_ESM.pdf]

nature research

Corresponding author(s): Alex K. Shalek

Last updated by author(s): July 12, 2021

Reporting Summary

Nature Research wishes to improve the reproducibility of the work that we publish. This form provides structure for consistency and transparency in reporting. For further information on Nature Research policies, see our Editorial Policies and the Editorial Policy Checklist.

Statistics

For all statistical analyses, confirm that the following items are present in the figure legend, table legend, main text, or Methods section.

n/a	Confirmed	
[ ]	[x]	The exact sample size (*n*) for each experimental group/condition, given as a discrete number and unit of measurement
[ ]	[x]	A statement on whether measurements were taken from distinct samples or whether the same sample was measured repeatedly
[ ]	[x]	The statistical test(s) used AND whether they are one- or two-sided<br>*Only common tests should be described solely by name; describe more complex techniques in the Methods section.*
[x]	[ ]	A description of all covariates tested
[ ]	[x]	A description of any assumptions or corrections, such as tests of normality and adjustment for multiple comparisons
[ ]	[x]	A full description of the statistical parameters including central tendency (e.g. means) or other basic estimates (e.g. regression coefficient) AND variation (e.g. standard deviation) or associated estimates of uncertainty (e.g. confidence intervals)
[ ]	[x]	For null hypothesis testing, the test statistic (e.g. *F*, *t*, *r*) with confidence intervals, effect sizes, degrees of freedom and *P* value noted<br>*Give P values as exact values whenever suitable.*
[x]	[ ]	For Bayesian analysis, information on the choice of priors and Markov chain Monte Carlo settings
[x]	[ ]	For hierarchical and complex designs, identification of the appropriate level for tests and full reporting of outcomes
[ ]	[x]	Estimates of effect sizes (e.g. Cohen's *d*, Pearson's *r*), indicating how they were calculated

Our web collection on statistics for biologists contains articles on many of the points above.

Software and code

Policy information about availability of computer code

Data collection	Images were collected using MetaMorph software (Molecular Devices, Sunnyvale, CA). Active illumination was controlled through Metamorph software and iQ3 software (Andor Technology, Oxford Instruments). FACS data analysis and sorting was performed using the Sony SH800Z system (Sony Biotechnology, Inc.). NMR samples examined at 400 MHz (proton resonance) were done so on instruments running Bruker TopSpin 2.1 (Billerica, MA). Samples examined at 600 MHz (proton resonance) were done so on instruments running TopSpin 3.5 (Billerica, MA). Mass spectrometry data was acquired using MassHunter Workstation Software – Qualitative Analysis Version B.03.01; build 3.1.346.6; service pack 2 (Agilent Technologies).
Data analysis	Cell Profiler v 3.1.9 was used to segment microscopy images, and resulting data was analyzed using MATLAB R2020a. Sequencing data was demultiplexed using bl2fastq v2.17.1.14. Smart-Seq2 sequencing reads were aligned using STAR v2.5b and transcripts were quantified with RSEM v1.2.3. Seq-Well sequencing reads were aligned and processed according to the Drop-Seq Computational Protocol v2.0 (https://github.com/broadinstitute/Drop-seq). Single-cell RNA-seq analysis was performed in R version 4.0.2 using ggplot2 (v3.3.2130), Seurat (v4.0.1), and ComplexHeatmap (v2.7.3132). Gene ontologies were evaluated for enrichment using DAVID v6.8. Additional NMR processing was done with MestReNova (9.0.1) (Santiago de Compostela, Spain). Mass spectrometry data was processed using MassHunter Workstation Software – Qualitative Analysis Version B.03.01; build 3.1.346.6; service pack 2 (Agilent Technologies).

For manuscripts utilizing custom algorithms or software that are central to the research but not yet described in published literature, software must be made available to editors and reviewers. We strongly encourage code deposition in a community repository (e.g. GitHub). See the Nature Research guidelines for submitting code & software for further information.

Data

Policy information about availability of data

All manuscripts must include a data availability statement. This statement should provide the following information, where applicable:

- Accession codes, unique identifiers, or web links for publicly available datasets
- A list of figures that have associated raw data
- A description of any restrictions on data availability

Source data are provided with this paper. The raw data and gene expression matrices for mouse tumor scRNA-seq data have been deposited in the Gene Expression Omnibus (https://www.ncbi.nlm.nih.gov/geo/query/acc.cgi?acc=GSE175882). For human intestinal organoids, interactive visualization tools, metadata, and digital gene expression matrices can be found through the Broad Institute's Single-Cell Portal as study SCP1457 (https://singlecell.broadinstitute.org/single_cell/study/SCP1457/spacecat-intestinal-organoid). FASTQ data for the human intestinal organoids is available upon request and with a data use agreement. Digital gene expression matrices annotated with cell types, photoactivation regions, and other metadata can also be found in Supplementary Data 1 (human intestinal organoids, corresponding to Fig. 2) and Supplementary Data 3 (mouse KP lung tumors, corresponding to Fig. 4). Gene lists corresponding to differential expression tests for human intestinal organoids and mouse KP lung tumors can be found in Supplementary Data 2 and 4, respectively.

Field-specific reporting

Please select the one below that is the best fit for your research. If you are not sure, read the appropriate sections before making your selection.

[x] Life sciences [ ] Behavioural & social sciences [ ] Ecological, evolutionary & environmental sciences

For a reference copy of the document with all sections, see nature.com/documents/nr-reporting-summary-flat.pdf

Life sciences study design

All studies must disclose on these points even when the disclosure is negative.

Sample size	Sample sizes for in vitro cell line photoactivation and sequencing were estimated based on necessary photoactivated area to include sufficient cells for 1 plate of plate-based scRNA-seq. Sample sizes for organoid photoactivation and sequencing were estimated to meet the goal of having >100 recovered photoactivated cells, given typical cell losses during scRNA-seq experimental workflows (as described in text). Sample sizes for tumor photoactivation and sequencing were determined by mouse model availability and tumor burden.
Data exclusions	A complementary 'core' sample from Tumor 1 in the KP model analysis was removed due to low cell recovery and a lack of a replicate in the second Tumor (Tumor 2). Cell pre-filtering is described in detail in methods for each scRNA-seq experiment; in brief, cells were filtered for common quality metrics like minimum number of detected genes, minimum detected UMIs and maximum percent of UMIs corresponding to mitochondrial genes. Cells that were removed due to quality metrics are not included in data available in the Supplementary Data files or public repositories.
Replication	Figure legends contain all details on replicate experiments and sample sizes for presented data, and all attempts at replication were successful. Photoactivation experiments on cell lines and tissue sections were performed and replicated across multiple donors, species, tissue-of-origin, and experimental days. Photoactivation experiments on organoids were replicated across multiple organoids in distinct culture wells.
Randomization	Wells of cells/organoids and tissue sections were randomly assigned to photoactivation or control condition to avoid batch effects.
Blinding	Researchers could not be blinded, as photoactivation required inspection and drawing of regions of interest.

Reporting for specific materials, systems and methods

We require information from authors about some types of materials, experimental systems and methods used in many studies. Here, indicate whether each material, system or method listed is relevant to your study. If you are not sure if a list item applies to your research, read the appropriate section before selecting a response.

Materials & experimental systems

n/a	Involved in the study
[ ]	[x] Antibodies
[ ]	[x] Eukaryotic cell lines
[x]	[ ] Palaeontology and archaeology
[ ]	[x] Animals and other organisms
[ ]	[x] Human research participants
[x]	[ ] Clinical data
[x]	[ ] Dual use research of concern

Methods

n/a	Involved in the study
[x]	[ ] ChIP-seq
[ ]	[x] Flow cytometry
[x]	[ ] MRI-based neuroimaging

Antibodies

Antibodies used	Alexa Fluor 647 anti-mouse TCR beta chain, clone H57-597, Biolegend, cat #109218
Validation	Manufacturer validated for IHC, quality tested for flow cytometry. (https://www.biolegend.com/en-us/products/alexa-fluor-647-anti-mouse-tcr-beta-chain-antibody-3272)

Eukaryotic cell lines

Policy information about cell lines

Cell line source(s)	Murine NIH/3T3 cells (ATCC, CRL-1658), human HEK293T cells (ATCC, CRL-11268)
Authentication	Cell lines were not authenticated.
Mycoplasma contamination	All cell lines tested negative for mycoplasma contamination.
Commonly misidentified lines (See ICLAC register)	No commonly misidentified cell lines were used in this study.

Animals and other organisms

Policy information about studies involving animals; ARRIVE guidelines recommended for reporting animal research

Laboratory animals	Genetically engineered mice were generated by injecting low passage Kras-Lox-Stop-Lox-G12D/+; p53fl/fl (KP) embryonic stem cells from the C57BL/6 background into albino C57BL/6 blastocysts. Both male and female animals were used, all were aged to 12 weeks prior to tumor initiation. All animals were housed in static caging, hardwood chip bedding, RO water, with irradiated chow (Ancare cages) with a 12/12 light/dark light cycle. Temperature was set to 70F with an acceptable range +/- 2 degrees with a humidity range of 30-70%.
Wild animals	The study did not involve wild animals.
Field-collected samples	The study did not involve samples collected from the field.
Ethics oversight	All studies were performed under animal protocols approved by the Harvard Medical School and Massachusetts Institute of Technology (MIT) Institutional Animal Care and Use Committees (IACUC).

Note that full information on the approval of the study protocol must also be provided in the manuscript.

Human research participants

Policy information about studies involving human research participants

Population characteristics	Population characteristics are not applicable as results were provided as proof-of-principle that the method would work on complex systems that are difficult to manipulate genetically. Further, a single donor's intestinal organoids were used, from de-identified human bulk surgical resections.
Recruitment	Small intestinal crypts were isolated as previously described from de-identified human bulk surgical resections.
Ethics oversight	All studies were performed under protocols approved by the Massachusetts Institute of Technology (MIT) Committee on the Use of Humans as Experimental Subjects and the Institutional Review Board (IRB) protocols of Massachusetts General Hospital/Partners Healthcare.

Note that full information on the approval of the study protocol must also be provided in the manuscript.

Flow Cytometry

Plots

Confirm that:

- [x] The axis labels state the marker and fluorochrome used (e.g. CD4-FITC).
- [x] The axis scales are clearly visible. Include numbers along axes only for bottom left plot of group (a 'group' is an analysis of identical markers).
- [x] All plots are contour plots with outliers or pseudocolor plots.
- [x] A numerical value for number of cells or percentage (with statistics) is provided.

Methodology

Sample preparation	Cultured cells and human intestinal organoids (HEK293T, NIH/3T3) were digested using Trypsin-LE (Life Technologies). The

cells were incubated with Trypsin-LE at 37C for 5 minutes, followed by the addition of complete media (DMEM + 10% FBS). Murine organs were digested using the gentleMACS Tissue Dissociation Kit (Miltenyi Biotec) according to manufacturer's instructions.

Instrument	Sony SH800Z
Software	Sony SH800Z analysis software
Cell population abundance	Quantitative values for flow cytometry/sorting abundances are included in each relevant figure.
Gating strategy	We took an expanded FSC/SSC gating to account for all cells with diverse sizes. Cells were gated first for viability based on calcein violet (DAPI channel), followed by calcein NVOC (FITC channel). Positive and negative gates are defined in each relevant plot, and are based on cells exposed to calcein NVOC, but not photo-activated using near-UV light.

☒ Tick this box to confirm that a figure exemplifying the gating strategy is provided in the Supplementary Information.
