# Supplementary material for: Live cell tagging tracking and isolation for spatial transcriptomics using photoactivatable cell dyes
Source: Nat Commun. 2021 Aug 17;12:4995. doi: 10.1038/s41467-021-25279-y (PMC8371137; doi:10.1038/s41467-021-25279-y)
Supplement: Supplementary file 7 — Description of Additional Supplementary Files [file 41467_2021_25279_MOESM7_ESM.pdf]

Title: Supplementary Data 1.
Description: Digital gene expression matrix and metadata for cells derived from human intestinal organoids.

Title: Supplementary Data 2.
Description: Differentially expressed genes between photoactivated and non-photoactivated control "Stem.1" cells.

Title: Supplementary Data 3.
Description: Digital gene expression matrix and metadata for cells derived from mouse KP lung tumors.

Title: Supplementary Data 4.
Description: Differentially expressed genes by cell type and tumor location in KP autochthonous lung tumors.
